# OVOL guides the epithelial-hybrid-mesenchymal transition

**DOI:** 10.18632/oncotarget.3623

**Published:** 2015-04-22

**Authors:** Dongya Jia, Mohit Kumar Jolly, Marcelo Boareto, Princy Parsana, Steven M. Mooney, Kenneth J. Pienta, Herbert Levine, Eshel Ben-Jacob

**Affiliations:** ^1^ Center for Theoretical Biological Physics, Rice University, Houston, TX 77005-1827, USA; ^2^ Graduate Program in Systems, Synthetic and Physical Biology, Rice University, Houston, TX 77005-1827, USA; ^3^ Department of Bioengineering, Rice University, Houston, TX 77005-1827, USA; ^4^ Department of Physics and Astronomy, Rice University, Houston, TX 77005-1827, USA; ^5^ Department of Biosciences, Rice University, Houston, TX 77005-1827, USA; ^6^ School of Physics and Astronomy and The Sagol School of Neuroscience, Tel-Aviv University, Tel-Aviv 69978, Israel; ^7^ Institute of Physics, University of Sao Paulo, Sao Paulo 05508, Brazil; ^8^ Department of Computer Science, Johns Hopkins University, Baltimore, MD 21287, USA; ^9^ The James Buchanan Brady Urological Institute, Johns Hopkins University School of Medicine, Baltimore, MD 21287, USA; ^10^ Department of Urology, Johns Hopkins University School of Medicine, Baltimore, MD 21287, USA; ^11^ Department of Oncology, Johns Hopkins University School of Medicine, Baltimore, MD 21287, USA; ^12^ Department of Pharmacology and Molecular Sciences, Johns Hopkins University School of Medicine, Baltimore, MD 21287, USA

**Keywords:** EMT, metastasis, OVOL, partial EMT, cancer systems biology

## Abstract

Metastasis involves multiple cycles of Epithelial-to-Mesenchymal Transition (EMT) and its reverse-MET. Cells can also undergo partial transitions to attain a hybrid epithelial/mesenchymal (E/M) phenotype that has maximum cellular plasticity and allows migration of Circulating Tumor Cells (CTCs) as a cluster. Hence, deciphering the molecular players helping to maintain the hybrid E/M phenotype may inform anti-metastasis strategies. Here, we devised a mechanism-based mathematical model to couple the transcription factor OVOL with the core EMT regulatory network miR-200/ZEB that acts as a three-way switch between the E, E/M and M phenotypes. We show that OVOL can modulate cellular plasticity in multiple ways - restricting EMT, driving MET, expanding the existence of the hybrid E/M phenotype and turning both EMT and MET into two-step processes. Our theoretical framework explains the differences between the observed effects of OVOL in breast and prostate cancer, and provides a platform for investigating additional signals during metastasis.

## INTRODUCTION

Metastasis, the cause of more than 90% of cancer-related deaths [1], begins when cancer cells go through an Epithelial-to-Mesenchymal Transition (EMT) to leave the primary tumor and migrate towards blood vessels. Next, the metastatic cells stay in blood circulation as Circulating Tumor Cells (CTCs) until they exit at distant organs to seed micrometastases. During seeding, cells undergo the reverse of EMT - Mesenchymal-to-Epithelial Transitions (MET) - to regain their epithelial characteristics and form secondary tumors [2]. Similar EMT-MET cycles also happen during embryonic development and tissue repair, but not in adult homeostasis. Their aberrant activation is a hallmark of cancer metastasis. Therefore, understanding this phenotypic plasticity of cancer cells is likely to provide important clues for hindering metastatic progression.

Transitions between epithelial and mesenchymal phenotypes may occur through an intermediate phenotype - hybrid epithelial/mesenchymal (E/M) phenotype [2, 3]. This hybrid E/M phenotype has been observed in tissue morphogenesis (type I EMT) [4], wound healing (type II EMT) [5], and in during metastasis (or type III) EMT [6, 7], and is considered to be ‘metastable’ [8]. It provides the cells with the maximum plasticity to switch to being epithelial or mesenchymal, thereby facilitating subsequent rounds of EMT and MET during organogenesis and the spread of metastatic disease [9]. Cells belonging to this phenotype have combined epithelial (cell-cell adhesion) and mesenchymal (motility) traits that enable them to migrate collectively, as seen during migration of CTCs as clusters in the bloodstream of lung, prostate and breast cancer patients [10–13]. These CTC clusters can have up to 50-times more metastatic potential than the individually migrating CTCs [14]. Presumably, seeding several cells at the new niche increases the probability of the micrometastases to develop into mature metastases [15]. Therefore, the ability of metastatic cells to acquire a hybrid E/M phenotype renders them to pose a higher risk of metastasis. Hence, deciphering the molecular players that enable the cells to maintain the hybrid E/M phenotype may inform anti-metastasis strategies.

Whether epithelial cells would undergo no EMT, partial EMT or complete EMT depends on the tissue-specific signaling pathways that regulate EMT/MET. In many carcinomas, these signals converge into a decision-making gene regulatory circuit that comprises of a mutually inhibitory feedback loop between the microRNA family miR-200 and the transcription factor family ZEB. Epithelial cells have high expression of miR-200 and low expression of ZEB, and conversely, mesenchymal cells have high expression of ZEB and low expression of miR-200 [16–18]. Recently, it was shown that the miR-200/ZEB circuit can operate as a three-way switch, allowing for an additional phenotype, hybrid E/M, that corresponds to medium expression of both miR-200 and ZEB [19, 20]. Notably, this epithelial-mesenchymal plasticity is regulated by tissue-specific coupling of this loop to many other key players.

One of the key players that affects EMT/MET in a tissue-specific context is a family of transcription factors- OVOL [21,22]. OVOL is a well-known regulator of embryogenesis [23–26] that is activated by BMP7/Smad4 pathway and C/EBP-β [27, 28], and inhibited by the repressor complex Armadillo/dTCF downstream of Wg signaling [25]. In prostate and breast cancer cell lines, OVOL can induce the expression of miR-200 and consequently acts as a driver of MET [29]. In a different context where EMT happens, during mammary morphogenesis, OVOL is expressed in the terminal end bud (TEB) cells that migrate collectively with finger-like projections, and its knockdown can lead to a complete EMT reflected by individual cell migration [22]. Thus, OVOL operates as a “critical molecular brake on EMT” by preventing the “TEB cells that have gained partial plasticity” from undergoing complete EMT, thus maintaining the cells in the hybrid E/M phenotype [22]. Together, these studies demonstrate that OVOL shepherds or guides the cell-fate determination, but it remains elusive how can it act as both a driver of MET and a brake holder of EMT.

**Figure 1:**
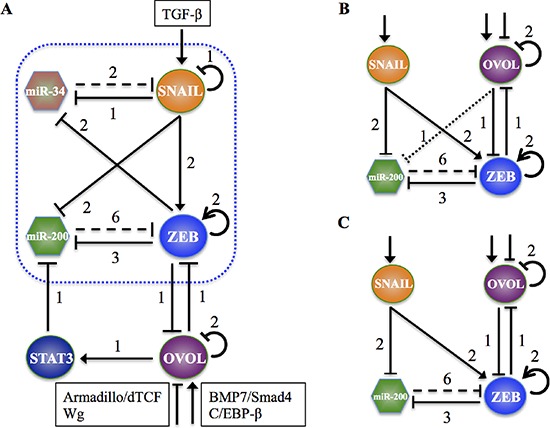
The regulatory network coupling OVOL with miR-200/ZEB **A.** The regulatory network coupling OVOL with miR-34/SNAIL and miR-200/ZEB circuits for prostate cancer. The part of the circuit in the dotted box shows the core EMT regulatory network. OVOL forms a mutually inhibitory loop with ZEB, inhibits miR-200 indirectly via STAT3 and is also self-inhibitory. TGF-β activates SNAIL, and BMP7/Smad4 pathway and C/EBP-β activate OVOL, but Wg signaling (Armadillo/dTCF) inhibits OVOL. The coupling of miR-34/SNAIL with miR-200/ZEB does not change the qualitative behavior of miR-200/ZEB circuit [19]. For this reason, in order to simplify the calculations, we didn't consider here the miR-34/SNAIL loop and simply treated SNAIL as an external signal on the miR-200/ZEB/OVOL circuit. **B.** Effective miR-200/ZEB/OVOL circuit for the case of prostate cancer, where OVOL inhibits both miR-200 and ZEB. **C.** Effective miR-200/ZEB/OVOL circuit for the case of breast cancer, where OVOL inhibits ZEB but not miR-200. A solid arrow denotes transcriptional activation, and a solid bar denotes transcriptional inhibition. Dashed line indicates microRNA-mediated translational regulation, and dotted line indicates indirect inhibition. The number listed along each line represents the number of binding sites on the promoter region of target genes (See SI section I for details). In B and C, external signals on SNAIL and OVOL denote those shown in A.

Understanding this shepherding role of OVOL calls for investigating the interplay of OVOL with the EMT regulatory circuit – miR-200/ZEB. OVOL and ZEB inhibit each other transcriptionally [29] and OVOL is self-inhibitory [30]. Also, in prostate cancer, but not breast cancer, OVOL activates STAT3 [21] that inhibits miR-200 [31] (Figure 1A–1C). Here, through mathematical modeling, we elucidate the role of OVOL in affecting cellular decision making between the acquisition of the three phenotypes – E, M and E/M. Given the different couplings of OVOL with miR-200/ZEB for prostate cancer and breast cancer, we analyze both these cases separately.

We found that in the presence of OVOL, higher levels of EMT-inducing signals (such as TGF-β) are required to induce a partial or complete EMT, because endogenous levels of OVOL maintain cells in epithelial and hybrid E/M phenotypes, hence acting as a brake holder of complete EMT. Consistently, inhibition of OVOL can drive a complete EMT. Also, OVOL enables higher epithelial-mesenchymal plasticity to the cells by allowing them to undergo a partial MET, and its overexpression can drive a complete MET. Therefore, the levels of OVOL can determine the phenotype that the cells adopt – epithelial, mesenchymal or hybrid E/M. Our results present OVOL as a transcription factor that shepherds EMT/MET – it can both drive MET and halt EMT in a context-dependent manner, and also plays a crucial role in helping cells maintain the hybrid E/M phenotype.

## RESULTS

### ZEB/OVOL coupling sets the conditions for phenotypic transitions

**Figure 2:**
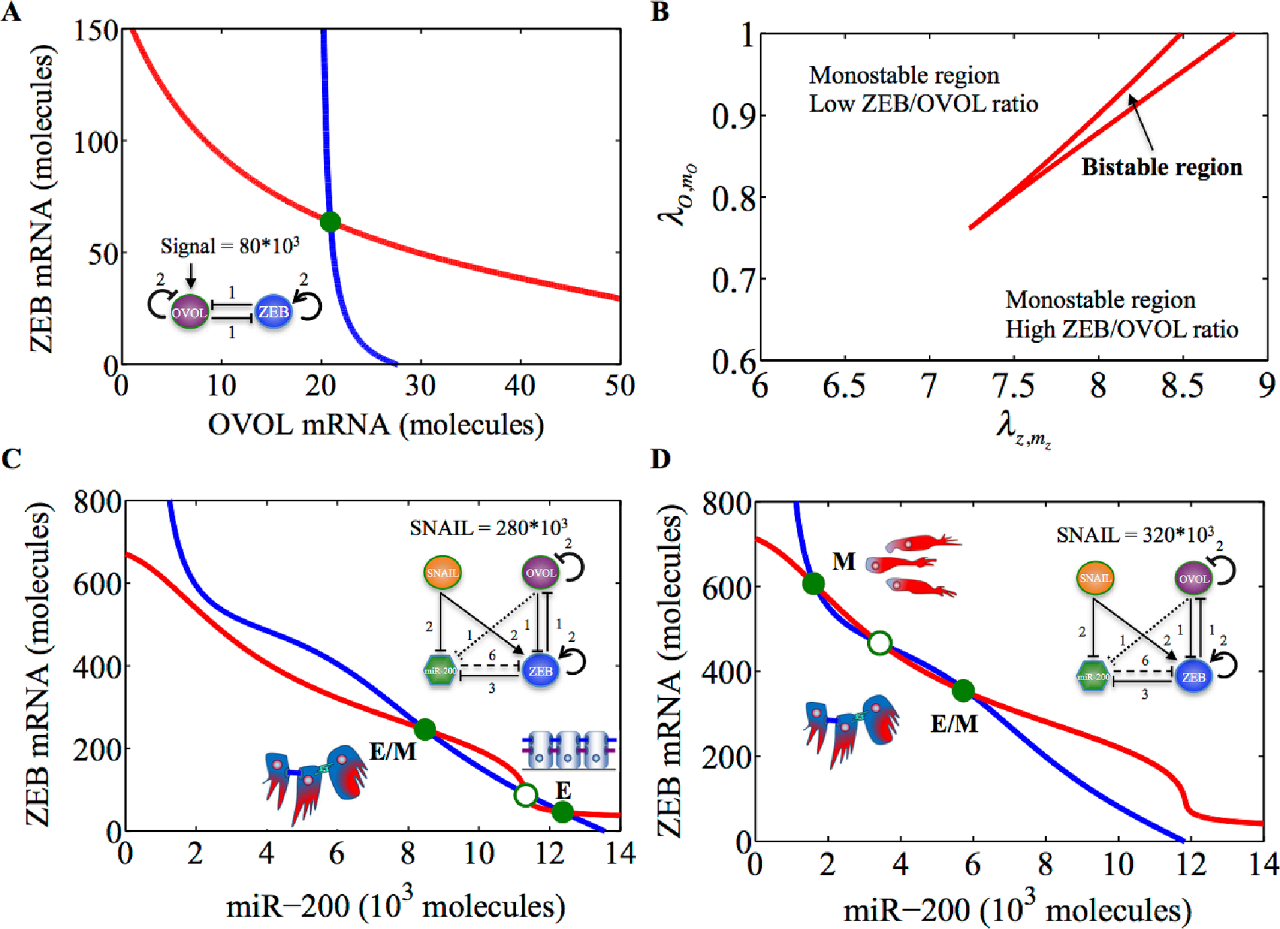
Nullclines of the miR-200/ZEB/OVOL module for prostate cancer **A.** Nullclines for ZEB/OVOL circuit for activation signal on OVOL at fixed level 80*10^3^ molecules. The circuit shows monostability. Red nullcline is for *dm_z_* / *dt* = 0, *dZ* / *dt* = 0, *dO* / *dt* = 0 and blue nullcline is for *dO* / *dt* = 0, *dm_o_* / *dt* = 0, *dZ* / *dt* = 0. **B.** Phase diagram of ZEB/OVOL circuit with regards to two control parameters - *λ_O,m_O__* (weight factor for OVOL self-inhibition that changes between 0 and 1) and *λ_Z,m_Z__* (weight factor for ZEB self-activation that increases from 1 till 10). The higher the value of *λ_Z,m_Z__*, the weaker the self-inhibition of OVOL, and the higher the value of *λ_O,m_O__*, the stronger the self-activation of ZEB. The area between the two red lines marks the parameter range for which ZEB/OVOL circuit is bistable. The axes here are zoomed in to show the region of bistability - x-axis varies from 6 to 9, and y-axis from 0.6 to 1. **C.** Nullclines of miR-200/ZEB/OVOL circuit (with the inhibition of miR-200 by OVOL) for fixed SNAIL levels = 280*10^3^ molecules. The possible stable steady states (phenotypes) are: E- (1, 0) or (high miR-200, low ZEB) with ZEB/OVOL ratio = 0.5 and E/M- (½, ½) or (medium miR-200, medium ZEB) with ZEB/OVOL ratio = 2.1. **D.** Nullcline of miR-200/ZEB/OVOL circuit (with the inhibition of miR-200 by OVOL) for fixed SNAIL levels = 320*10^3^ molecules. The possible phenotypes are: E/M - (½, ½) or (medium miR-200, medium ZEB) with ZEB/OVOL ratio = 2.1 and M- (0, 1) or (low miR-200, high ZEB) with ZEB/OVOL ratio = 16.7. For C and D, red nullcline is for *dZ* / *dt* = 0, *dm_Z_* / *dt* = 0, *dO* / *dt* = 0, *dm_O_* / *dt* = 0, blue nullcline is for *dO* / *dt* = 0, *dμ*_200_ / *dt* = 0, *dm_O_* / *dt* = 0, *dZ* / *dt* = 0. Green solid circles denote stable fixed points, and green hollow circles denote unstable fixed points. Corresponding phenotypes have been depicted alongside the stable steady states.

As a first step towards understanding the effect of OVOL on EMT/MET, we analyze the dynamics of the ZEB/OVOL mutual inhibition circuit (without including miR-200) driven by a fixed input signal I, mimicking the effects of signals that activate OVOL such as the BMP7/Smad4 pathway. We found that this circuit generically exhibits monostability, i.e. it cannot give rise to phenotypic transitions since only a single stable state (phenotype) can exist (Figure 2A). The term ‘generically’ indicates that it is the characteristics behavior of the circuit for a wide range of physiologically realistic parameters deduced from experiments (Table S1, 2). As the signal I increases, the expression values of the stable state shifts smoothly towards higher levels of OVOL and lower levels of ZEB. This shift allows the ratio of ZEB to OVOL protein levels (or ZEB/OVOL ratio) to vary along a continuum without undergoing any phenotypic transitions. Since ZEB can drive EMT [16, 17] and OVOL can drive MET (or halt EMT) [22, 29], the ZEB/OVOL ratio can serve as a trigger or threshold point for EMT and MET. The exact physiological parameters of the circuit depend on the genetic/epigenetic profile of cells in a particular cancer [29], therefore the ZEB/OVOL ratio required for phenotypic transitions (EMT or MET) is case-specific, thus explaining variations in epithelial plasticity.

Next, we investigate the effects of different strengths of the self-inhibition of OVOL and self-activation of ZEB on the ZEB/OVOL expression ratio. ZEB/OVOL circuit is monostable for a wide range of the circuit parameters, but it can give rise to bistability (give rise to phenotypic transitions) for very strong self-activation of ZEB and a very weak self-inhibition of OVOL (Figure 2B). This result suggests that the self-inhibition of OVOL, but not the self-activation of ZEB, plays a crucial role in maintaining the ZEB/OVOL circuit to be monostable.

Further, it is the OVOL self-inhibition, but not ZEB self-activation, that can reduce the impact of external noise in the incoming signals that activate OVOL (such as BMP4/Smad7) (Figure S3). This noise-buffering characteristic of OVOL can prevent aberrant activation of EMT due to transient inputs from the microenvironment, and can “safeguard epithelial identity” [32]. Such a “guardian” role for OVOL has been shown during epidermal differentiation and mammary duct elongation, cases where EMT needs to be repressed; and depleting OVOL interferes with proper epithelial differentiation [22, 32, 33].

Equipped with the new understanding, we proceeded to investigate the dynamics of the combined miR-200/ZEB/OVOL circuit driven by EMT-inducing signals (such as TGF-β) upstream of SNAIL. We find that this circuit acts as a three-way switch giving rise to three states (phenotypes): (i) Epithelial (E) phenotype (low ZEB, high miR-200), (ii) hybrid Epithelial/Mesenchymal (E/M) phenotype (medium ZEB, medium miR-200), and (iii) Mesenchymal (M) phenotype (low ZEB, high miR-200). These three phenotypes correspond to different ZEB/OVOL expression ratios (Figure 2C, 2D). At lower levels of EMT-inducing signals, this ratio is low, and consequently the cells can be either in the epithelial or hybrid E/M phenotype (Figure 2C). However, at higher levels of SNAIL, this ratio increases and the cells can undergo complete EMT (Figure 2D). These results are consistent irrespective of whether OVOL inhibits miR-200 or not (Figure 2, Figure S4), i.e. the ZEB/OVOL expression ratio regulates the transition point for both EMT and MET, for both breast cancer and prostate cancer.

### OVOL as a break holder of complete EMT and an expander of hybrid E/M phenotype

**Figure 3:**
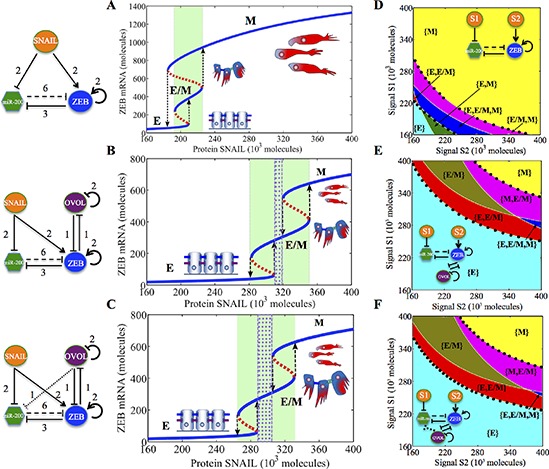
Dynamical system characteristics of the miR-200/ZEB/OVOL circuit **A.** Bifurcation of mRNA levels of ZEB in response to SNAIL levels for miR-200/ZEB circuit. It illustrates the possible co-existence (for some range of SNAIL levels) of the three possible stable states for same physiological conditions - E - (1, 0), E/M - (½, ½) and M - (0,1). **B.** Bifurcation of mRNA levels of ZEB in response to SNAIL levels for miR-200/ZEB/OVOL circuit (without the inhibition of miR-200 by OVOL). **C.** Bifurcation of mRNA levels of ZEB in response to SNAIL levels for miR-200/ZEB/OVOL circuit (with the inhibition of miR-200 by OVOL). Phase-diagram driven by two independent signals S1 and S2 representing SNAIL, as illustrated in the *Inset* circuit for **D.** miR-200/ZEB circuit, **E.** miR-200/ZEB/OVOL circuit (without the inhibition of miR-200 by OVOL), and **F.** miR-200/ZEB/OVOL circuit (with the inhibition of miR-200 by OVOL). Each phase (denoted by a different color) corresponds to a different combination of co-existing states or phenotypes. The bifurcation diagram and phase diagram in every row are for the circuit drawn in the leftmost column of that row. The region marked by purple dots in B, C represents the range of SNAIL levels for which the hybrid E/M phenotype can exist alone, and the region marked by green in (A), (B), (C), and that by black dots in (D), (E), (F) represents the range of SNAIL levels for which the hybrid E/M phenotype can exist alone or as one of the multiple possible phenotypes.

The response of the miR-200/ZEB/OVOL circuit to different levels of SNAIL (e.g. via TGF-β) is presented as a bifurcation diagram in Figure 3. For low SNAIL levels, cells attain the epithelial (E) phenotype, and as SNAIL increases, EMT is induced, however only partially, and the cells attain the hybrid E/M phenotype. Further increase in SNAIL levels induces a complete EMT, and the cells adopt a mesenchymal (M) phenotype. The range of values of SNAIL for which the hybrid E/M phenotype exists is larger for miR-200/ZEB/OVOL circuit as compared to that for miR-200/ZEB circuit in the absence of the OVOL, irrespective of whether OVOL inhibits miR-200 or not (compare the green shaded region in Figures 3B and 3C vs. that in Figure 3A). These results indicate that in presence of OVOL, higher levels of EMT-inducing signals are required to complete EMT.

These results are consistent with the experimentally suggested role of OVOL in preventing the cells that have undergone partial EMT to undergo complete EMT, hence acting as a “critical molecular brake on EMT” [22]. Similarly, SNAIL levels required to induce even a partial EMT are higher for the case of miR-200/ZEB/OVOL circuit as compared to those for the miR-200/ZEB circuit. Hence, OVOL expands the range of physiological conditions for which the epithelial and hybrid E/M phenotypes can exist, thus halting the progression of EMT.

During MET, when SNAIL levels are decreased, mesenchymal cells initially undergo a partial MET to attain the hybrid E/M phenotype and on further decrease in SNAIL, MET is completed, i.e. the cells attain an epithelial phenotype (Figure 3B, 3C). This behavior is different from that of miR-200/ZEB circuit, where EMT happens through the hybrid E/M phenotype, but MET proceeds directly from mesenchymal phenotype to the epithelial one (Figure 3A). Therefore, OVOL facilitates higher plasticity on the epithelial-mesenchymal axis in both directions (EMT/MET).

To further investigate the role of OVOL in EMT/MET, a phase diagram (two-dimensional bifurcation diagram) where the action of SNAIL (or equivalently TGF- β) is represented by two independent signals – S1, a transcriptional activator of ZEB and S2, a transcriptional inhibitor of miR-200 – was calculated (Figure 3D–3F). These diagrams demonstrate multiple phases (sets of co-existing phenotypes for the same physiological conditions): three phases where cells can be in only one phenotype - {E}, {M} and {E/M}, three phases where cells can be in one of two possible phenotypes – {E, M}, {E/M, M} and {E, E/M}, and one phase where cells can be in one of three possible phenotypes – {E, M, E/M}. The phenotype selection, when more than one phenotype is possible, depends on the specific epigenetic and genetic profile of that individual cell, its local microenvironment and the previous signals it has received. Clearly, the total area corresponding to the phases that contain E/M as one of the states or the only state increases in presence of OVOL (compare the area bounded by black dots in Figures 3E, 3F vs. that in Figure 3D).

Previously, we found that without OVOL, the E/M phenotype can only exist when other phenotypes are also possible, i.e. in the {E/M, M}, {E/M, E} and {E, E/M, M} phases [19]. Here we found that in the presence of OVOL, it can also exist alone in {E/M} phase, i.e. it can be the only possible phenotype for a range of physiological parameters. This result holds true irrespective of whether OVOL inhibits miR-200 or not. In addition, the range of physiological parameters for which the hybrid E/M phenotype can exist alone or as one of multiple possible phenotypes increases when OVOL effect is included, and the effect is more pronounced in the case of prostate cancer (Figure 3A–3F).

Further, the inhibitory feedback of OVOL by ZEB has been reported for prostate and breast cancer [29], but not during mammary morphogenesis and epidermal development [22, 33]. Comparing these two situations (with and without the inhibitory feedback), we found that the effect of OVOL as a promoter of the hybrid E/M phenotypes is amplified when it is weakly or not inhibited by ZEB (Figure S5, S6). These results might explain why some studies interpreted OVOL as a “molecular brake on EMT” rather than a MET driver [22]. Hence, a plausible reason why OVOL might not be inhibited by ZEB during mammary morphogenesis and epidermal development is the need for higher plasticity.

Put together, the model investigations show that endogenous or basal levels of OVOL in the cells can extend the range of physiological conditions for which the hybrid E/M phenotype can exist and restrict the progression towards complete EMT in multiple contexts – breast and prostate cancer, mammary morphogenesis, and epidermal differentiation.

### Over-expression of OVOL can drive the mesenchymal-to-epithelial transition

**Figure 4:**
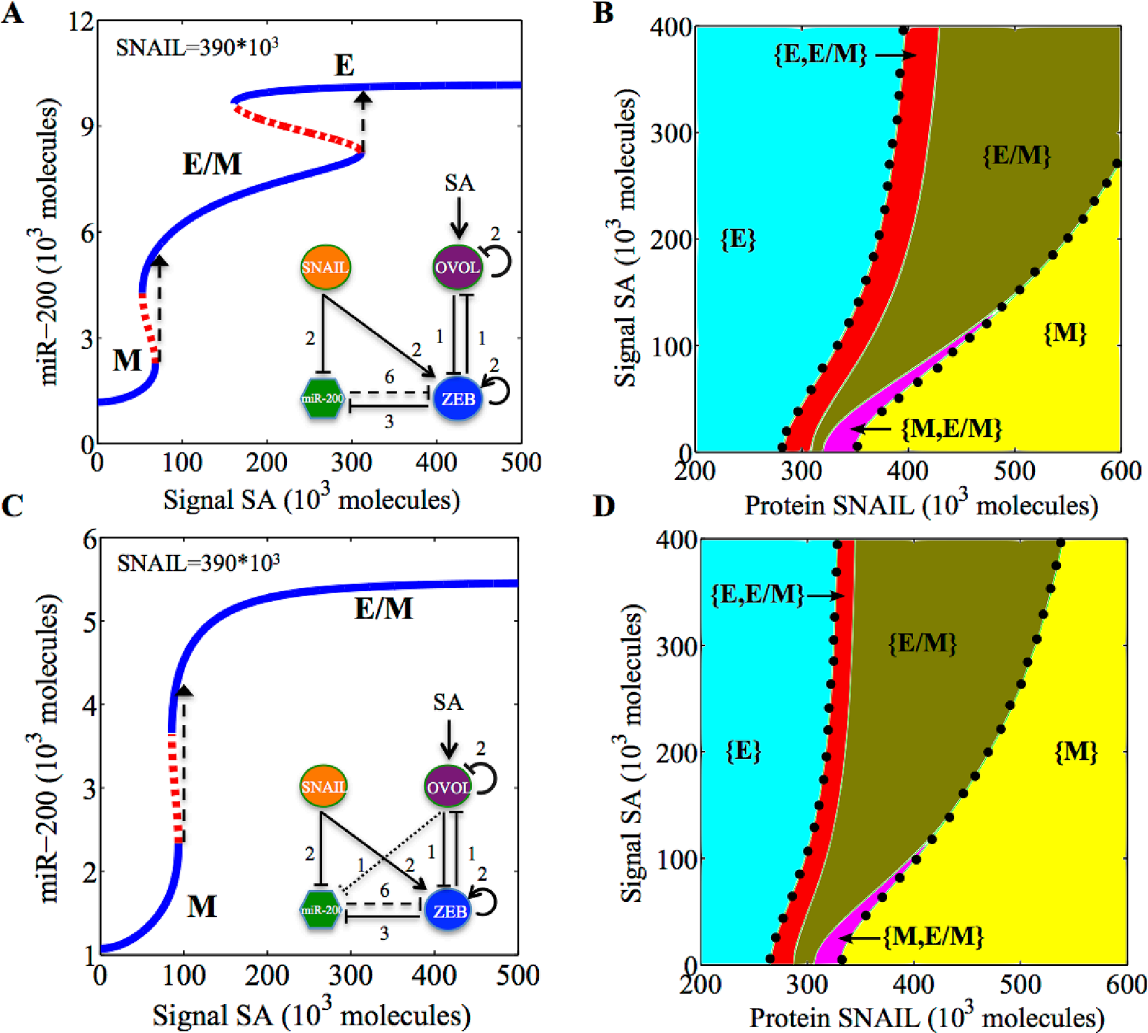
Bifurcation and phase diagram for the miR-200/ZEB/OVOL circuit driven by an external activation signal (SA) on OVOL A and B show the results for the miR-200/ZEB/OVOL circuit for the case of breast cancer (no inhibition of miR-200 by OVOL), C and D show those for the case of prostate cancer (with the inhibition of miR-200 by OVOL). **A.** Bifurcation of miR-200 levels when the cell is initially in mesenchymal or (0, 1) phenotype (as enabled by high SNAIL levels = 390 × 10^3^ molecules) and is driven by varying activation signal (SA) on OVOL. **B.** Phase diagram of the miR-200/ZEB/OVOL circuit when it is driven by two independent signals, SNAIL and (SA). **C.** Bifurcation of miR-200 levels when the cell is initially in mesenchymal or (0,1) phenotype (as enabled by high SNAIL levels= 390 × 10^3^ molecules) and is driven by a varying activation signal (SA) on OVOL. **D.** Phase-diagram of the miR-200/ZEB/OVOL when variable levels of both SNAIL and (SA) drive the circuit. Different colors in (B) and (D) represent different phases (or set of co-existing phenotypes for the same physiological conditions). Area bound by the black dots shows the total range of physiological parameters for which the hybrid E/M phenotype exists, either alone or in combination with other possible phenotypes.

Next, to mimic the effect of OVOL being activated by BMP7/Smad4 pathway and/or C/EBP-β [27, 28], we evaluate the effect of an external activation signal (SA) on OVOL under different levels of SNAIL. For both breast cancer and prostate cancer, a weak activation of OVOL causes the mesenchymal cells (i.e. cells with high levels of SNAIL) to undergo a partial MET to attain a hybrid E/M phenotype, even at high levels of SNAIL (Figure 4A, 4C). Stronger activation can cause the breast cancer cells in mesenchymal phenotype to go through complete MET and attain the epithelial phenotype (Figure 4A). These results, are consistent with the experimentally identified role of OVOL as an MET-inducer [29]. In the case of prostate cancer, due to the additional inhibition of miR-200 by OVOL, complete MET might not be induced in prostate cancer even at high levels of OVOL expression (Figure 4C). These results explain the experimental observations that overexpression of OVOL in mesenchymal prostate cancer cells (PC3-EMT14) increased the levels of miR-200 only modestly and the cells did not go through a complete MET [29].

For low levels of EMT-inducing signals, cells are in the partial EMT or hybrid E/M state for endogenous levels (no external activation) of OVOL; and a complete MET can be induced by overexpression of OVOL, both for breast cancer and for prostate cancer (Figure S7). Our results are consistent with experiments showing that the high expression of OVOL correlates with an epithelial phenotype, and reduces the migration and metastatic potential of breast and prostate cancer cells [29].

Further, we calculate a phase diagram where variable levels of both SNAIL and an external activation signal on OVOL (mimicking, for example, the effect of BMP7/Smad4 pathway) drive the miR-200/ZEB/OVOL circuit. We found that on increasing the activation signals on OVOL, the hybrid E/M phenotype can exist for a wider range of SNAIL, for both breast cancer and prostate cancer (Figure 4B, 4D).

### OVOL knockdown is critical for cells to complete EMT

**Figure 5:**
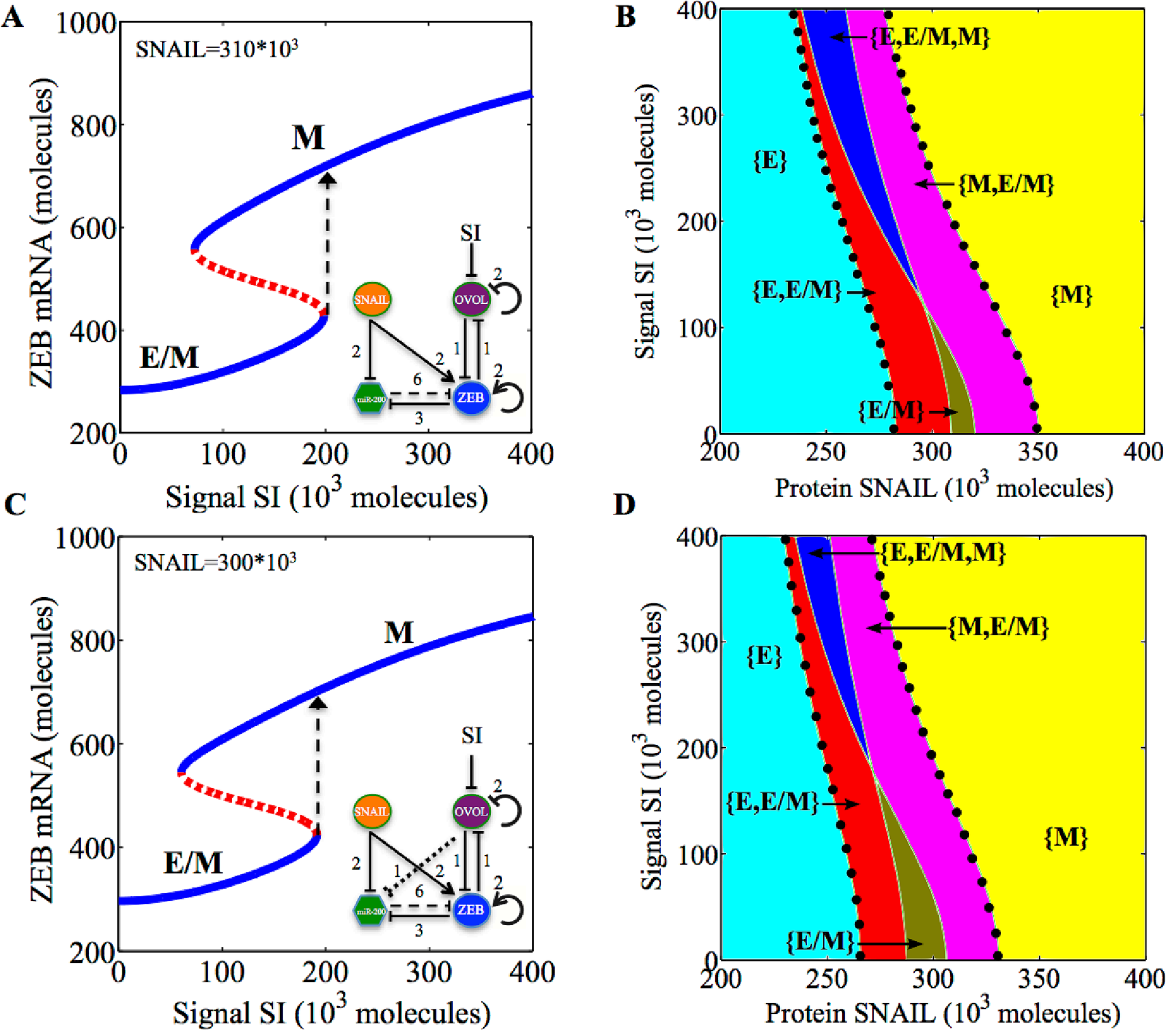
Bifurcation and phase diagram for miR-200/ZEB/OVOL circuit in response to an external inhibition signal (SI) on OVOL A and B show the results for the miR-200/ZEB/OVOL circuit for the case of breast cancer, C and D show those for the case of prostate cancer. **A.** Bifurcation of ZEB mRNA levels when the cell is initially in hybrid E/M phenotype (as enabled by SNAIL = 310*10^3^ molecules) and is driven by varying inhibition signal (SI) on OVOL. **B.** Phase diagram of miR-200/ZEB/OVOL circuit when it is driven by two independent signals, SNAIL and (SI). **C.** Bifurcation of ZEB mRNA levels when the cell is initially in hybrid E/M phenotype (as enabled by SNAIL = 300*10^3^ molecules) and is driven by a varying inhibition signal (SI) on OVOL. **D.** Phase-diagram of miR-200/ZEB/OVOL when variable levels of both SNAIL and (SI) drive the circuit. Different colors in (B) and (D) represent different phases. Area bound by the black dots shows the total range of physiological parameters for which the hybrid E/M phenotype exists, either alone or in combination with other possible phenotypes.

To investigate the effect of inhibition of OVOL, for example, by Armadillo/dTCF (Wg signaling), we evaluate the effect of an external inhibition signal (SI) on OVOL for different levels of EMT-inducing signal SNAIL. For epithelial and mesenchymal cells, inhibiting OVOL does not cause a phenotype transition (Figure S8). However, for cells in the hybrid E/M phenotype, inhibiting OVOL causes them to undergo a complete EMT for both prostate cancer and breast cancer (Figure 5A, 5C). These results suggest a modest effect of the Wg signaling on miR-200/ZEB/OVOL and highlight that OVOL needs to be knocked down for cells to complete the EMT, or that the “critical molecular brake on EMT” [22] needs to be lost to drive the cells to complete EMT.

Further, we explore the behavior of the miR-200/ZEB/OVOL circuit when driven by variable levels of SNAIL and an external inhibition signal of OVOL. We found that the inhibition of OVOL has only a weak effect on the range of existence of the hybrid E/M phenotype for breast cancer, prostate cancer as well as mammary morphogenesis (Figures 5B, 5D and S6). This result is corroborated with experiments showing that during mammary morphogenesis, knockdown of OVOL leads to individual cell migration (mesenchymal phenotype) [22].

### Temporal dynamics of epithelial-hybrid-mesenchymal transitions

**Figure 6:**
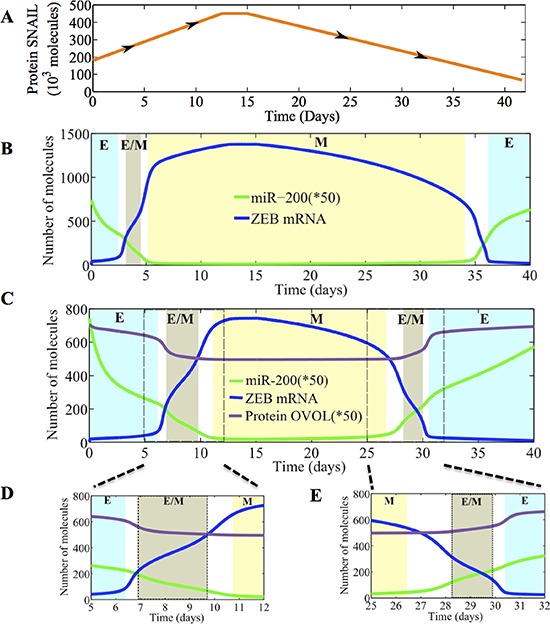
Temporal dynamics of epithelial-hybrid-mesenchymal transitions **A.** Time-varying external signal (SNAIL levels) applied to miR-200/ZEB/OVOL circuit (with the inhibition of miR-200 by OVOL). **B.** Temporal evolution of miR-200 (green, scaled by 0.02 to fit in the plot) and ZEB mRNA (blue) for the miR-200/ZEB module. This figure shows that EMT is a two-step process, E->E/M->M, while MET is a one-step process, from M-> E directly. **C.** Temporal evolution of miR-200 (green, scaled by 0.02 to fit in the plot), ZEB mRNA (blue) and protein OVOL (purple, scaled by 0.02 to fit in the plot) for the miR-200/ZEB/OVOL module. Areas shown in the boxes (days 5-12 and days 25-32) are expanded in **D.** and **E.** to show that the cells pass through the hybrid E/M state while undergoing EMT or MET. Different colors in B–E represent different stable states or phenotypes - cyan for E or (1,0) state, brown for hybrid E/M or (½, ½) state, yellow for M or (0,1) state.

To distinguish between the dynamics of EMT/MET for the miR-200/ZEB circuit and the miR-200/ZEB/OVOL circuit, we present their dynamical response to temporally varying levels of SNAIL for both prostate cancer (Figure 6) and breast cancer (Figure S9). We found that in the presence of OVOL, cells stay in the hybrid E/M phenotype for a longer duration (compare the width of the light brown rectangle in Figure 6C vs. that in Figure 6B). In addition, OVOL delays the onset of transition from epithelial to the E/M phenotype (compare the beginning time of the light brown rectangle in Figure 6C vs. that in Figure 6B). These diagrams also denote that OVOL enables the cells to undergo partial MET, a feature that is not observed for the miR-200/ZEB circuit without OVOL (two brown rectangles in Figure 6C vs. one in Figure 6B). Therefore, OVOL can render both EMT and MET to be two-step processes (E-E/M-M and M-E/M-E).

## DISCUSSION

Phenotypic transitions between epithelial and mesenchymal phenotypes (EMT and MET) play a crucial role in cancer metastasis and embryonic development [2, 34]. These transitions can happen through an intermediate or hybrid epithelial/mesenchymal (E/M) phenotype [2, 3]. The recognized importance of this hybrid E/M phenotype has led to intense experimental efforts [6, 7, 35–39], yet it has been given limited theoretical attention till date. Here, we reveal a novel role for the transcription factor family OVOL in shepherding or guiding partial and complete EMT/MET.

Our results show that OVOL allows higher epithelial-mesenchymal plasticity to cells by fine-tuning the ZEB/OVOL expression ratio and thus deciding the susceptibility of the cancer cells to undergo EMT or MET [29]. This susceptibility is likely to depend on the extent of negative correlation between ZEB and OVOL levels (Figure S11) that can be specific to the tumor type. Such plasticity may also be beneficial during cell-fate decision of neuroectoderm/mesendoderm, where OVOL and ZEB promote opposite fates [40, 41]. Also, the self-inhibition of OVOL prevents aberrant EMT/MET activation, and can be critical during mammary morphogenesis and epidermal development when EMT needs to be repressed [22, 33].

Further, we show that in prostate cancer, breast cancer as well as mammary morphogenesis, OVOL can extend the range of physiological conditions under which the hybrid E/M phenotype can exist. Preliminary data shows that OVOL can also transcriptionally inhibit SNAIL (unpublished, Pienta group). We show that this additional link allows more plasticity (Figure S10). Also, OVOL activates NF-kB [21] that plays a key role in associating the hybrid E/M phenotype with a high likelihood of gaining stemness [42]. Therefore, OVOL might be crucial in not only promoting the existence of the hybrid E/M phenotype, but also associating it with gaining stemness. However, future studies are required to validate this proposed role of OVOL.

An important prediction emerging from our analysis is that OVOL enables the cells to undergo a partial MET, i.e. in presence of OVOL, transitions from both E to M and M to E happen through the hybrid E/M phenotype – however, without OVOL, such a plasticity is possible only under the effect of biological noise [19]. With recent studies reporting partial MET during metastases [43], it would be important to explore the role of OVOL during colonization of CTCs when EMT needs to be suppressed and cells need to undergo a partial or complete MET [44, 45].

Our findings that the loss of OVOL can drive the cells to being mesenchymal are in agreement with experiments showing that knockdown of OVOL causes solitary cell migration [22], and that over-expression of OVOL reduces the migration of cancer cells significantly [29]. Further, one mechanism through which TGF-β drives EMT is by inhibiting C/EBP-β [46], an activator of OVOL [28] that also prevents EMT in tubular epithelial cells [47]. Besides, BMP7/Smad4 signaling pathway, an upstream activator of OVOL [27], also counteracts TGF-β driven EMT in renal tubular epithelial cells as well as mammary ductal epithelial cells [48]. The model presented here explains how these diverse observations fit together.

In summary, we present the first step towards understanding the role of transcription factor family OVOL in regulating both forward and backward epithelial-hybrid-mesenchymal transitions. Future efforts should investigate the role of OVOL in modulating other key cellular properties associated with EMT such as stemness [42], drug resistance [49] and senescence [50]. With increasing attempts being made to map and quantitatively understand cancer signaling networks [51–57], the theoretical framework presented here can serve as a basis for future incorporation of additional signals such as p53, TGF-β and HIF-1α, Notch signaling, to elucidate their effects on epithelial-mesenchymal plasticity. A better understanding and control of this plasticity holds promise to provide valuable clues for future development of improved therapeutic strategies to target metastasis [34].

## METHODS

### Mathematical model formulation

There are five components in the miR-200/ZEB/OVOL module - microRNA miR-200 (*μ*_200_), ZEB mRNA (*m_Z_*), ZEB protein (*Z*), OVOL mRNA (*m_O_*), and OVOL protein (*O*). All these species have an innate production and degradation rate. Transcriptional regulation is denoted by shifted Hill functions (*H^S+^* for transcriptional activation and *H^S-^* for transcriptional inhibition. Details of shifted Hill functions can be found in SI section 1). To capture the effects of miRNA, we consider both the degradation of mRNA by miRNAs (depicted by *Y_m_*) and the inhibition of translation by miRNAs (depicted by *L*). Also, the miRNAs that bind to mRNAs can be degraded after forming a complex with mRNAs (depicted by *Y_μ_*).

For the case of prostate cancer (OVOL inhibits both miR-200 and ZEB (Figure 1B)), the dynamics of miR-200 (*μ*_200_) can be described by the following equation:

(1) $$\frac{d\mu_{200}}{dt}=g_{\mu_{200}}H^{S-}\left(O,\lambda_{O,\mu_{200}}\right)H^{S-}\left(Z,\lambda_{Z,\mu_{200}}\right)H^{S-}\left(S,\lambda_{S,\mu_{200}}\right)-m_{Z}Y_{\mu }\left(\mu_{200}\right)-k_{\mu_{200}}\mu_{200}$$

where *g*_*μ*_200__ and *k*_*μ*_200__ are innate production and degradation rates of miR-200 respectively. *H^S−^*(*O*, *λ*_*O,μ*_200__) represents the transcriptional inhibition of miR-200 by protein OVOL, *H^S−^*(*Z*, *λ*_*Z,μ*_200__) represents the transcriptional inhibition of miR-200 by protein ZEB and *H^S−^*(*S*, *λ*_*S,μ*_200__) represents the transcriptional inhibition of miR-200 by protein SNAIL. *Y_μ_*(*μ*_200_) represents the degradation of miR-200 caused by forming complex with ZEB mRNAs.

Dynamics of ZEB mRNA (*m_Z_*) and ZEB protein (*Z*) is described by the following equations:

(2) $$\frac{dm_{Z}}{dt}=g_{m_{Z}}H^{S-}\left(O,\;\lambda_{O,m_{Z}}\right)H^{S+}\left(Z,\;\lambda_{Z,m_{Z}}\right)H^{S+}\left(S,\;\lambda_{S,m_{Z}}\right)-m_{Z}Y_{m}\left(\mu_{200}\right)-k_{m_{Z}}m_{Z}$$

(3) $$\frac{dZ}{dt}=g_{Z}m_{Z}L\left(\mu_{200}\right)-k_{Z}Z$$

where *g_m_Z__* and *g_z_* are the innate production rates of ZEB mRNA and ZEB protein respectively, and *k_m_Z__* and *k_z_* are their respective innate degradation rates. *H^S−^*(*O, λ*_*O,m_Z_*_) represents the transcriptional inhibition of ZEB mRNA by protein OVOL, *H*^*S*+^(*Z, λ*_*Z,m_Z_*_) denotes the transcriptional self-activation of ZEB mRNA, and *H*^*S*+^(*S, λ*_*S,m_Z_*_) denotes the transcriptional activation of ZEB mRNA by protein SNAIL. *Y_m_*(*μ*_200_) represents the degradation of ZEB mRNA by forming complex with miR-200, and *L*(*μ*_200_) denotes the translational inhibition of ZEB mRNA by miR-200.

Dynamics of OVOL mRNA (*m_o_*) and protein (*O*) is described by the following equations:

(4) $$\frac{dm_{O}}{dt}=g_{m_{O}}H^{S-}\left(O,\lambda_{O,m_{O}}\right)H^{S-}\left(Z,\lambda_{Z,m_{O}}\right)-k_{m_{O}}m_{O}$$

(5) $$\frac{dO}{dt}=g_{O}m_{O}-k_{O}O$$

where *g_m_O__* and *g_O_* are the innate production rates of OVOL mRNA and OVOL protein respectively, *k_m_O__* and *k_o_* are their respective degradation rates. *H^S−^*(*O, λ*_*O,m_O_*_) represents the transcriptional self-inhibition of OVOL mRNA, and *H^S−^*(*Z, λ*_*Z,m_O_*_) represents the transcriptional inhibition of OVOL mRNA by protein ZEB. To incorporate the effect of an external activation and inhibition signal on OVOL, we multiply an extra shifted Hill function to *g_mo_* in equation (4).

For the case of breast cancer (OVOL inhibits only ZEB and not miR-200), the term *H^S−^*(*O, λ*_*O,μ*_200__) is omitted in equation (1).

Later, we incorporated the effect of BMP7/Smad4 pathway that activates OVOL and that of Armadillo/dTCF complex that inhibits OVOL. Details of the model construction and parameter values used in the model can be found in SI section 1 (Table S1, S2). The model is quite robust with respect to changes in parameter values as discussed in SI section 2 (Figure S1 for prostate cancer and Figure S2 for breast cancer).

## SUPPLEMENTARY INFORMATION FIGURES AND TABLES



## References

[1] Gupta GP, Massagué J (2006). Cancer metastasis: building a framework. Cell.

[2] Nieto MA (2013). Epithelial plasticity: a common theme in embryonic and cancer cells. Science.

[3] Revenu C, Gilmour D (2009). EMT 2.0: shaping epithelia through collective migration. Curr Opin Genet Dev.

[4] Micalizzi DS, Farabaugh SM, Ford HL (2010). Epithelial-mesenchymal transition in cancer: parallels between normal development and tumor progression. J Mammary Gland Biol Neoplasia.

[5] Arnoux V, Côme C, Kusewitt DF, Hudson LG, Savagner P (2005). Cutaneous Wound Reepithelialization. Rise and Fall of Epithelial Phenotype. Springer US.

[6] Huang RY-J, Wong MK, Tan TZ, Kuay KT, Ng a HC, Chung VY (2013). An EMT spectrum defines an anoikis-resistant and spheroidogenic intermediate mesenchymal state that is sensitive to e-cadherin restoration by a src-kinase inhibitor, saracatinib (AZD0530). Cell Death Dis.

[7] Sampson VB, David JM, Puig I, Patil PU, de Herreros AG, Thomas G V, Rajasekaran AK (2014). Wilms' tumor protein induces an epithelial-mesenchymal hybrid differentiation state in clear cell renal cell carcinoma. PLoS One.

[8] Lee JM, Dedhar S, Kalluri R, Thompson EW (2006). The epithelial-mesenchymal transition: new insights in signaling, development, and disease. J Cell Biol.

[9] Thiery JP, Acloque H, Huang RYJ, Nieto MA (2009). Epithelial-mesenchymal transitions in development and disease. Cell.

[10] Yu M, Bardia A, Wittner BS, Stott SL, Smas ME, Ting DT (2013). Circulating breast tumor cells exhibit dynamic changes in epithelial and mesenchymal composition. Science.

[11] Lecharpentier A, Vielh P, Perez-Moreno P, Planchard D, Soria JC, Farace F (2011). Detection of circulating tumour cells with a hybrid (epithelial/mesenchymal) phenotype in patients with metastatic non-small cell lung cancer. Br J Cancer.

[12] Hou J-M, Krebs M, Ward T, Sloane R, Priest L, Hughes A, Clack G, Ranson M, Blackhall F, Dive C (2011). Circulating Tumor Cells as a Window on Metastasis Biology in Lung Cancer. Am J Pathol.

[13] Armstrong AJ, Marengo MS, Oltean S, Kemeny G, Bitting RL, Turnbull JD, Herold CI, Marcom PK, George DJ, Garcia-Blanco MA (2011). Circulating tumor cells from patients with advanced prostate and breast cancer display both epithelial and mesenchymal markers. Mol cancer Res MCR.

[14] Aceto N, Bardia A, Miyamoto DT, Donaldson MC, Wittner BS, Spencer JA (2014). Circulating tumor cell clusters are oligoclonal precursors of breast cancer metastasis. Cell.

[15] Ben-Jacob E, Coffey DS, Levine H (2012). Bacterial survival strategies suggest rethinking cancer cooperativity. Trends Microbiol.

[16] Burk U, Schubert J, Wellner U, Schmalhofer O, Vincan E, Spaderna S, Brabletz T (2008). A reciprocal repression between ZEB1 and members of the miR-200 family promotes EMT and invasion in cancer cells. EMBO Rep.

[17] Bracken CP, Gregory PA, Kolesnikoff N, Bert AG, Wang J, Shannon MF, Goodall GJ (2008). A double-negative feedback loop between ZEB1-SIP1 and the microRNA-200 family regulates epithelial-mesenchymal transition. Cancer Res.

[18] Gregory PA, Bert AG, Paterson EL, Barry SC, Tsykin A, Farshid G, Vadas Ma, Khew-Goodall Y, Goodall GJ (2008). The miR-200 family and miR-205 regulate epithelial to mesenchymal transition by targeting ZEB1 and SIP1. Nat Cell Biol.

[19] Lu M, Jolly MK, Levine H, Onuchic JN, Ben-Jacob E (2013). MicroRNA-based regulation of epithelial-hybrid-mesenchymal fate determination. Proc Natl Acad Sci U S A.

[20] Lu M, Jolly MK, Onuchic J, Ben-Jacob E (2014). Toward Decoding the Principles of Cancer Metastasis Circuits. Cancer Res.

[21] Roca H, Pande M, Huo JS, Hernandez J, Cavalcoli JD, Pienta KJ, McEachin RC (2014). A bioinformatics approach reveals novel interactions of the OVOL transcription factors in the regulation of epithelial - mesenchymal cell reprogramming and cancer progression. BMC Syst Biol.

[22] Watanabe K, Villarreal-Ponce A, Sun P, Salmans ML, Fallahi M, Andersen B, Dai X (2014). Mammary morphogenesis and regeneration require the inhibition of EMT at terminal end buds by Ovol2 transcriptional repressor. Dev Cell.

[23] Dai X, Schonbaum C, Degenstein L, Bai W, Mahowald A, Fuchs E (1998). The ovo gene required for cuticle formation and oogenesis in flies is involved in hair formation and spermatogenesis in mice. Genes Dev.

[24] Mackay DR, Hu M, Li B, Rheaume C, Dai X (2006). The mouse Ovol2 gene is required for cranial neural tube development. Dev Biol.

[25] Payre F, Vincent A, Carreno S (1999). ovo/svb integrates Wingless and DER pathways to control epidermis differentiation. Nature.

[26] Nair M, Teng A, Bilanchone V, Agrawal A, Li B, Dai X (2006). Ovol1 regulates the growth arrest of embryonic epidermal progenitor cells and represses c-myc transcription. J Cell Biol.

[27] Kowanetz M, Valcourt U, Bergstrom R, Heldin C-H, Moustakas A (2004). Id2 and Id3 Define the Potency of Cell Proliferation and Differentiation Responses to Transforming Growth Factor? and Bone Morphogenetic Protein. Mol Cell Biol.

[28] Gomis RR, Alarcon C, He W, Wang Q, Seoane J, Lash A, Massague J (2006). A FoxO-Smad synexpression group in human keratinocytes. Proc Natl Acad Sci U S A.

[29] Roca H, Hernandez J, Weidner S, McEachin RC, Fuller D, Sud S (2013). Transcription Factors OVOL1 and OVOL2 Induce the Mesenchymal to Epithelial Transition in Human Cancer. PLoS One.

[30] Nair M, Bilanchone V, Ortt K, Sinha S, Dai X (2007). Ovol1 represses its own transcription by competing with transcription activator c-Myb and by recruiting histone deacetylase activity. Nucleic Acids Res.

[31] Guo L, Chen C, Shi M, Wang F, Chen X, Diao D, Hu M, Yu M, Qian L, Guo N (2013). Stat3-coordinated Lin-28-let-7-HMGA2 and miR-200-ZEB1 circuits initiate and maintain oncostatin M-driven epithelial-mesenchymal transition. Oncogene.

[32] Li S, Yang J (2014). Ovol Proteins: Guardians against EMT during Epithelial Differentiation. Dev Cell.

[33] Lee B, Villarreal-Ponce A, Fallahi M, Ovadia J, Sun P, Yu Q-C, Ito S, Sinha S, Nie Q, Dai X (2014). Transcriptional mechanisms link epithelial plasticity to adhesion and differentiation of epidermal progenitor cells. Dev Cell.

[34] Pinto CA, Widodo E, Waltham M, Thompson EW (2013). Breast cancer stem cells and epithelial mesenchymal plasticity - Implications for chemoresistance. Cancer Lett.

[35] Futterman MA, García AJ, Zamir E a (2011). Evidence for partial epithelial-to-mesenchymal transition (pEMT) and recruitment of motile blastoderm edge cells during avian epiboly. Dev Dyn.

[36] Schliekelman MJ, Taguchi A, Zhu J, Dai X, Rodriguez J, Celiktas M (2015). Molecular portraits of epithelial, mesenchymal and hybrid states in lung adenocarcinoma and their relevance to survival. Cancer Res.

[37] Leroy P, Mostov KE (2007). Slug Is Required for Cell Survival during Partial Epithelial-Mesenchymal Transition of HGF-induced tubulogenesis. J Cell Sci.

[38] Jordan NV, Johnson GL, Abell AN (2011). Tracking the intermediate stages of epithelial-mesenchymal transition in epithelial stem cells and cancer. Cell Cycle.

[39] GarcÃa de Herreros A (2014). Epithelial to mesenchymal transition in tumor cells as consequence of phenotypic instability. Front Cell Dev Biol.

[40] Zhang T, Zhu Q, Xie Z, Chen Y, Qiao Y, Li L, Jing N (2013). The zinc finger transcription factor Ovol2 acts downstream of the bone morphogenetic protein pathway to regulate the cell fate decision between neuroectoderm and mesendoderm. J Biol Chem.

[41] Chng Z, Teo A, Pedersen RA, Vallier L (2010). SIP1 Mediates Cell-Fate Decisions between Neuroectoderm and Mesendoderm in Human Pluripotent Stem Cells. Cell Stem Cell.

[42] Jolly MK, Huang B, Lu M, Mani SA, Levine H, Ben-jacob E (2014). Towards elucidating the connection between epithelial – mesenchymal transitions and stemness. J R Soc Interface.

[43] Chao Y, Wu Q, Acquafondata M, Dhir R, Wells A (2011). Partial Mesenchymal to Epithelial Reverting Transition in Breast and Prostate Cancer Metastases. Cancer Microenviron.

[44] Ocaña OH, Córcoles R, Fabra A, Moreno-Bueno G, Acloque H, Vega S, Barrallo-Gimeno A, Cano A, Nieto MA (2012). Metastatic colonization requires the repression of the epithelial-mesenchymal transition inducer Prrx1. Cancer Cell.

[45] Tsai JH, Donaher JL, Murphy DA, Chau S, Yang J (2012). Spatiotemporal regulation of epithelial-mesenchymal transition is essential for squamous cell carcinoma metastasis. Cancer Cell.

[46] Johansson J, Berg T, Kurzejamska E, Pang M-F, Tabor V, Jansson M, Roswall P, Pietras K, Sund M, Religa P, Fuxe J (2013). MiR-155-mediated loss of C/EBPβ shifts the TGF-β response from growth inhibition to epithelial-mesenchymal transition, invasion and metastasis in breast cancer. Oncogene.

[47] Xie P, Sun L, Nayak B, Haruna Y, Liu F, Kashihara N, Kanwar YS (2009). C/EBP-β Modulates Transcription of Tubulointerstitial Nephritis Antigen in Obstructive Uropathy. J Am Soc Nephrol.

[48] Zeisberg M, Hanai J, Sugimoto H, Mammoto T, Charytan D, Strutz F, Kalluri R (2003). BMP-7 counteracts TGF-beta1-induced epithelial-to-mesenchymal transition and reverses chronic renal injury. Nat Med.

[49] Oliveras-Ferraros C, Corominas-Faja B, Vazquez-Martin SA, Martin-Castillo B, Iglesias JM, López-Bonet E, Martin ÁG, Menendez JA (2012). Epithelial-to-mesenchymal transition (EMT) confers primary resistance to trastuzumab (Herceptin). Cell Cycle.

[50] Smit MA, Peeper DS (2010). Epithelial-mesenchymal transition and senescence: Two cancer-related processes are crossing paths. Aging (Albany NY).

[51] Balázsi G, van Oudenaarden A, Collins JJ (2011). Cellular decision making and biological noise: from microbes to mammals. Cell.

[52] Steinway SN, Gomez Tejeda Zañudo J, Ding W, Rountree CB, Feith DJ, Loughran TP, Albert R (2014). Network modeling of TGFβ signaling in hepatocellular carcinoma epithelial-to-mesenchymal transition reveals joint Sonic hedgehog and Wnt pathway activation. Cancer Res.

[53] Huang B, Lu M, Jolly MK, Tsarfaty I, Onuchic J, Ben-Jacob E (2014). The three-way switch operation of Rac1/RhoA GTPase-based circuit controlling amoeboid-hybrid-mesenchymal transition. Sci Rep.

[54] Chung S, Cooper CR, Farach-Carsron MC, Ogunnaike BA (2012). A control engineering approach to understanding the TGF- b paradox in cancer. J R Soc Interface.

[55] Boareto M, Jolly MK, Lu M, Onuchic JN, Clementi C, Ben-Jacob E (2015). Jagged-Delta asymmetry in Notch signaling can give rise to a Sender/Receiver hybrid phenotype. Proc Natl Acad Sci.

[56] Lin K, Baritaki S, Militello L, Malaponte G, Bevelacqua Y, Bonavida B (2010). The Role of B-RAF Mutations in Melanoma and the Induction of EMT via Dysregulation of the NF-kB/Snail/RKIP/PTEN Circuit. Genes Cancer.

[57] Lu M, Jolly MK, Gomoto R, Huang B, Onuchic J, Ben-Jacob E (2013). Tristability in Cancer-Associated MicroRNA-TF Chimera Toggle Switch. J Phys Chem B.

