# Supplementary material for: OVOL guides the epithelial-hybrid-mesenchymal transition
Source: Oncotarget. 2015 Apr 22;6(17):15436–48. doi: 10.18632/oncotarget.3623 (PMC4558162; doi:10.18632/oncotarget.3623)
Supplement: Supplementary file 1 [file oncotarget-06-15436-s001.pdf]

SUPPLEMENTARY INFORMATION

Circuit construction, model formulation and parameters used

Figure 1A is a schematic diagram of the EMT regulatory network coupling OVOL with miR-200/ZEB for prostate cancer. The circuit marked in the dotted box is the core EMT regulatory network that includes two highly interconnected modules – mutual inhibitory circuits between miR-34/SNAIL and miR-200/ZEB. The miR-34/SNAIL circuit behaves as a monostable noise-buffering integrator and miR-200/ZEB has been shown as the three-way switch that acts as decision-making circuit for cells to undergo partial or complete EMT [1]. Coupling miR-34/SNAIL with miR-200/ZEB (i.e. including the feedback inhibition of miR-34 by ZEB) doesn't change the three-way switch behavior of miR-200/ZEB [1]. Therefore, we didn't include miR-34/SNAIL loop in our analysis here and treated SNAIL levels as an external signal for the miR-200/ZEB circuit, as shown in Figure 1B and 1C.

The miR-200 family includes two subgroups based on seed sequences, miR-141 and miR-200a (Group I), and miR-200c and miR-429 (Group II). The ZEB family includes two members, ZEB1 and ZEB2. There are eight (three for group I and five for group II) conserved binding sites for miR-200 on the 3'UTR of ZEB1, and nine (three for group I and six for group II) on the 3' UTR of ZEB2. Both ZEB1 and ZEB2 bind to the conserved sites in the promoter regions of miR-200 family [2]. Since expression of E-cadherin can be restored by the stable expression of miR-200c alone [3], we consider six binding sites of miR-200 family to ZEB family, and three binding sites of ZEB family to miR-200 family. ZEB also can promote its own transcription through stabilizing SMAD complexes [4], we assume that ZEB self-activates through binding to two sites in the promoter region. SNAIL can activate the transcription of ZEB [5] and inhibit the expression of miR-200 [6] by binding to the E-boxes on the promoter regions. We assume both miR-200 and ZEB have two binding sites for SNAIL.

OVOL transcription factor family has two members - OVOL1 and OVOL2. OVOL2 binds directly to one consensus site in ZEB1's promoter and transcriptionally represses it [7]. Also, the knockout of ZEB induced the expression of both OVOL1 and OVOL2 [7]. Here, we have assumed that ZEB inhibits OVOL expression by binding to one site in its promoter region. Also, OVOL inhibits its own transcription both directly and indirectly [8], therefore we assume that OVOL self-inhibits by binding to two binding sites in the promoter region; and our assumption is validated by CHIP-Seq data investigating OVOL binding sites (unpublished, Pienta group; see section S11).

We describe the effect of transcriptional regulation of one species Y by the other X by using shifted Hill functions that are defined as $H^S(X,\lambda_{X,Y}) = H^-(X) + \lambda_{X,Y}H^+(X)$, where $H^+(X)$ is the excitatory Hill function, $H^-(X)$ is the inhibitory Hill function, and the weight factor $\lambda_{X,Y}$ denotes the fold-change in production rate of Y due to the regulation by X [1]. Therefore, for transcriptional activation, $\lambda_{X,Y}$ >1; for transcriptional repression, $\lambda_{X,Y}$ <1; and if there is no transcriptional regulation between X and Y, $\lambda_{X,Y} = 1$. More specifically, $H^{S-}(X,\lambda_{X,Y})$ represents the transcriptional inhibition (i.e. $\lambda_{X,Y}$ <1), and $H^{S+}(X,\lambda)$ represents transcriptional activation (i.e. $\lambda_{X,Y}$ >1).

The effect of microRNA-based regulation is described by Y (degradation of miRNA and mRNA due to miRNA-mRNA complex formation) and L (translational inhibition) functions. In the microRNA-based regulation model, one or more microRNAs (miRNA, depicted by μ) can bind to the 3'UTR of the target mRNA (depicted by m) to form a miRNA-mRNA complex. miRNAs can inhibit the translation of mRNA and/or degrade mRNA, and can be degraded or recycled. Involving all these possible effects, we use $L(\mu) = \sum_{i=0}^{n} l_i C_n^i M_n^i(\mu)$ to denote the translational inhibition, $Y_m(\mu) = \sum_{i=0}^{n} \gamma_{mi} C_n^i M_n^i(\mu)$ to denote the degradation of mRNA due to the mRNA-miRNA complex and $Y_\mu(\mu) = \sum_{i=0}^{n} i\gamma_{\mu i} C_n^i M_n^i(\mu)$ to denote the degradation of the miRNA due to the mRNA-miRNA complex, where $M_n^i(\mu) = \frac{(\mu/\mu_0)^i}{(1+\mu/\mu_0)^n}$ and $\sum_{i=0}^{n} C_n^i M_n^i(\mu) = 1$. l_i, γ_{mi}, $\gamma_{\mu i}$ represent the individual translation rate of mRNA, individual degradation rate for mRNA and individual degradation rate for miRNA respectively (values given in table SI2). μ_0 represents the miRNA threshold that has been chosen to be 10000 molecules. Here, we suppose there are n miRNA binding sites on mRNA, hence there are totally $(n+1)$ possible configurations of mRNA, which can be bound by 0~n miRNAs. C_n^i is the number of combinations of i miRNAs on n miRNA binding sites, defined as $\frac{n!}{i!(n-i)!}$. Details about the derivation of L, Y_m and Y_μ functions can be found in our previous work [1].

The translation rate for one gene is around 140 proteins per mRNA per hour [9], so we used 100 proteins per ZEB mRNA and 200 proteins per OVOL mRNA as their respective translation rates. The innate degradation rates of miRNAs, mRNAs and proteins were selected based on their half-lives from experimental data. Typically, the half-life of mammalian proteins is about 10 hours [10]; therefore we selected 0.1 hour^{-1} as the innate degradation rate for protein ZEB and protein OVOL. The half-life of mRNA is a few hours [11], so we chose 0.5 hour^{-1} as the innate degradation rate for ZEB mRNA and OVOL

mRNA. And the innate degradation rate of miR-200 was selected as 0.05 hour^{-1} since generally miRNA is more stable than mRNA [12,13].

Estimation of expression levels for miRNAs, mRNAs and proteins were according to their typical concentrations in eukaryotic cells. Typically, the mammalian cell volume is 100-10000 um^3 and the concentration for a single protein is 10nM-1uM [14]. For 1uM protein, the number of protein molecules is $6.02 \times 10^{23} \times 10^{-6} \times (10000 \times (10^{-5})^3)$, i.e. around 6 million. Moreover, the ratio of protein/mRNA of one gene is about 2800 [9], so the number of mRNA for one gene should be of the order of 1000 molecules. The number of microRNA is about 10000 molecules [15].

With respect to the transcriptional regulation, the changes in synthesis rates from basal levels have been considered from two-fold up to ten-fold, i.e. the λ for activator ranges from 5 to 10, the λ for repressor from 0.5 to 0.1. The full list of parameters corresponding to shifted Hill functions is listed in Table S1, and those for L and Y functions in Table S2.

Sensitivity analysis

To understand the sensitivity of our predictions to the parameters listed above, we conducted a sensitivity analysis by varying every parameter one at a time by ± 20 % of its original value. The numbers of binding sites have been considered to be fixed. All other parameters – production and degradation rates, thresholds and weight factors of the shifted Hill functions – have been varied.

Coupling OVOL with miR-200/ZEB enlarges the range of SNAIL levels for the existence of hybrid E/M region (Figure 3). We first analyze the miR-200/ZEB/OVOL circuit for prostate cancer (where OVOL inhibits both ZEB and miR-200). In our sensitivity analysis, we increased or decreased each parameter by 20% (each case is denoted by an alphanumeric code) and plotted the range of SNAIL levels for which the hybrid E/M phenotype exists (Figure S1). The absolute levels of SNAIL that enable the existence of hybrid E/M state increase or decrease for changes in most parameters. However, here we focus on the changes in the range of SNAIL levels

Supplementary Table S1: List of parameters for the shifted Hill functions

Description	Fold change	Value	Number of binding sites	Value	Threshold	(molecules)
Self-inhibition of OVOL	λ_{O,m_O}	0.1	n_{O,m_O}	2	$O^0_{m_o}$	25000
Inhibition of OVOL by ZEB	λ_{Z,m_O}	0.5	n_{Z,m_O}	1	$Z^0_{m_O}$	10000
Self-activation of ZEB	λ_{Z,m_Z}	7.5	n_{Z,m_Z}	2	$Z^0_{m_z}$	25000
Inhibition of ZEB by OVOL	λ_{O,m_Z}	0.1	n_{O,m_Z}	1	$O^0_{m_Z}$	25000
Inhibition of miR-200 by OVOL	$\lambda_{O,\mu_{200}}$	0.1	$n_{O,\mu_{200}}$	1	$O^0_{\mu_{200}}$	250000
Inhibition of miR-200 by ZEB	$\lambda_{Z,\mu_{200}}$	0.1	$n_{Z,\mu_{200}}$	3	$Z^0_{\mu_{200}}$	220000
Activation of ZEB by SNAIL	λ_{S,m_Z}	10	n_{S,m_Z}	2	$S^0_{m_z}$	180000
Inhibition of miR-200 by SNAIL	$\lambda_{S,\mu_{200}}$	0.1	$n_{S,\mu_{200}}$	2	$S^0_{\mu_{200}}$	180000
External activation signal on OVOL	λ_{SA,m_O}	10	n_{SA,m_O}	2	SA_{0,m_O}	180000
External inhibition signal on OVOL	λ_{SI,m_O}	0.1	n_{SI,m_O}	2	SI_{0,m_O}	180000

Supplementary Table S2: Parameters of l_i, γ_{mi}, $\gamma_{\mu i}$, as used in the L, Y_m and Y_μ functions

n (number of miRNA binding sites)	0	1	2	3	4	5	6
l_i (hour^{-1})	1	0.6	0.3	0.1	0.05	0.05	0.05
γ_{mi} (hour^{-1})	0	0.04	0.2	1	1	1	1
$\gamma_{\mu i}$(hour^{-1})	0	0.005	0.05	0.5	0.5	0.5	0.5

for the existence of hybrid E/M state, when different parameters are varied. Compared with the control case (no parameter changed – case 0 in FigureS1A–S1C), the range of SNAIL for the existence of E/M state decreases when the strength of ZEB activation by SNAIL is increased (case E1), strength of ZEB self-activation is increased (case F1) (Figure S1A), threshold for ZEB self-activation is increased (case P1), threshold of ZEB levels for shifted Hill function representing inhibition of ZEB on miR-200 are decreased (case Q2) (Figure S1B), the innate production rate of ZEB mRNA is increased (case U1) and the innate degradation rate of ZEB mRNA is decreased (case Z2) (Figure S1C). All these cases, except for case P1, represent cases where the effective ZEB levels that can inhibit miR-200 and drive EMT are increased, i.e. the propensity of the cell to undergo EMT is increased. Also, consistently, at increased ZEB levels, OVOL levels are decreased (ZEB inhibits OVOL) and hence the effect of OVOL in expanding the hybrid E/M range is decreased. Conversely, the parameter changes that are likely to decrease the effective ZEB levels that can induce EMT should enlarge the range of SNAIL levels for which the hybrid E/M state exists. This is indeed observed for cases when the strength of ZEB activation by SNAIL is decreased (case E2), strength of ZEB self-activation is decreased (case F2) (Figure S1A), threshold of ZEB levels for shifted Hill function representing inhibition of ZEB on miR-200 are increased (case Q1) (Figure S1B), and the innate production rate of ZEB mRNA is decreased (case U2) (Figure S1C). Therefore, change of several parameters of the miR-200/ZEB circuit, especially those affecting the ZEB protein levels, can affect the range of SNAIL levels for which hybrid E/M state exists. The change of parameters with respect to OVOL didn't affect this range.

A similar sensitivity analysis was also conducted for the miR-200/ZEB/OVOL circuit for the case of breast cancer (where OVOL inhibits ZEB, but not miR-200) (Figure S2A–S2C). Similar to the case of prostate cancer, the range of SNAIL levels for the existence of hybrid E/M phenotype is most severely affected by parameters that tend to increase levels of ZEB and therefore weaken the effect of OVOL - when the strength of ZEB activation by SNAIL is increased (case D1), strength of ZEB self-activation is increased (case E1) (Figure S2A), threshold for the self-activation of ZEB (case N2), threshold of ZEB levels for shifted Hill function representing inhibition of ZEB on miR-200 are decreased (case O2) (Figure S2B), the innate production rate of ZEB mRNA is increased and (case S1) and the innate degradation rate is decreased (Figure S2C). However, as an exception, this range also decreases for the case of threshold for ZEB self-activation being increased (case P1), i.e. effective ZEB levels being curtailed. On the other hand, this range increases most significantly in those cases that tend to decrease ZEB levels, and hence increase the OVOL levels and consequently the role of OVOL in expanding the hybrid E/M phenotype. The change of parameters with respect to OVOL didn't affect this range largely.

Therefore, Figure S1 and S2 show that for most parameter changes (either increase or decrease), the region of SNAIL levels for which the hybrid E/M exists doesn't change much, as long as the ZEB levels were not very high, thereby suggesting that our prediction regarding the role of OVOL is quite robust to parameter variations.

Figure S1A represent the cases for 20% increase and decrease in the weight factors for different shifted Hill functions that represent fold-changes from the basal synthesis rate due to transcriptional regulation. A1 and A2 represent the case for 20% increase and decrease in fold-change for the inhibition of miR-200 by OVOL (depicted by $\lambda_{O,\mu_{200}}$). B1 and B2 represent the case for 20% increase and decrease in fold-change for the self-inhibition of OVOL (depicted by λ_{O,m_O}). C1 and C2 represent the case for 20% increase and decrease in fold-change for the inhibition of OVOL by ZEB (depicted by λ_{Z,m_O}). D1 and D2 represent the case for 20% increase and decrease in fold-change parameter for the inhibition of ZEB by OVOL (depicted by λ_{O,m_Z}). E1 and E2 represent the case for 20% increase and decrease in fold change for the activation of ZEB by SNAIL (depicted by λ_{S,m_Z}). F1 and F2 represent the case for 20% increase and decrease in fold change parameter for the self-activation of ZEB (depicted by λ_{Z,m_Z}). G1 and G2 represent the case for 20% increase and decrease in fold change parameter for the inhibition of miR-200 by SNAIL (depicted by $\lambda_{S,\mu_{200}}$). H1 and H2 represent the case for 20% increase and decrease in the fold change parameters for the inhibition of miR-200 by ZEB (depicted by $\lambda_{Z,\mu_{200}}$).

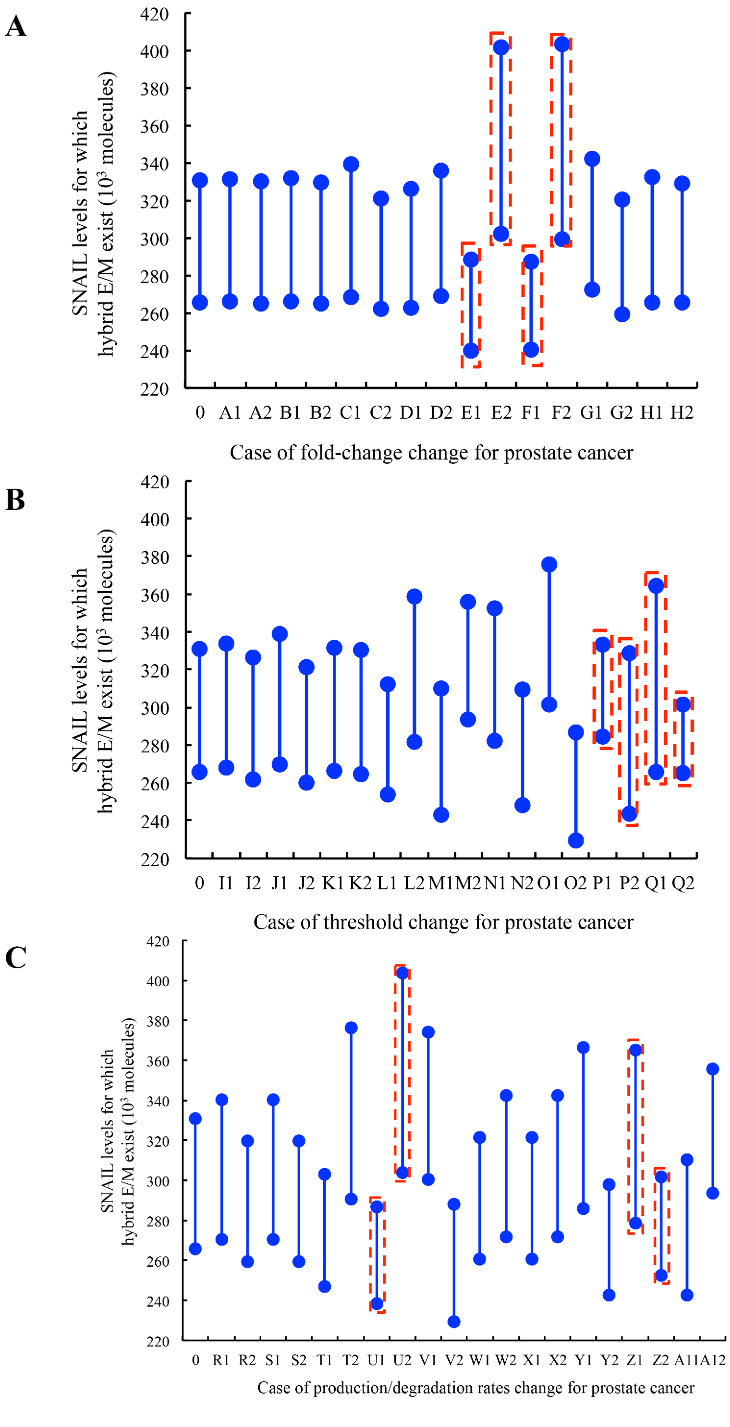

Supplementary Figure S1: Sensitivity analysis of miR-200/ZEB/OVOL circuit driven by SNAIL for prostate cancer. Alphanumeric codes on the x-axis represent the cases of different changed parameters. 0 represents the case for parameters in Figure 3C (control case).

Figure S1B represent the cases for 20% increase and decrease in the threshold levels of the different shifted Hill functions. I1 and I2 represent the case for 20% increase and decrease in threshold levels of OVOL for miR-200 inhibition (depicted by $O_0^{\mu_{200}}$). J1 and J2 represent the case for 20% increase and decrease in threshold levels of OVOL for its self-inhibition (depicted by $O_0^{m_O}$). K1 and K2 represent the case for 20% increase and decrease in threshold levels of ZEB for OVOL inhibition (depicted by $Z_0^{m_O}$). L1 and L2 represent the case for 20% increase and decrease in threshold levels of OVOL for ZEB inhibition (depicted by $O_0^{m_Z}$). M1 and M2 represent the case for 20% increase and decrease in threshold

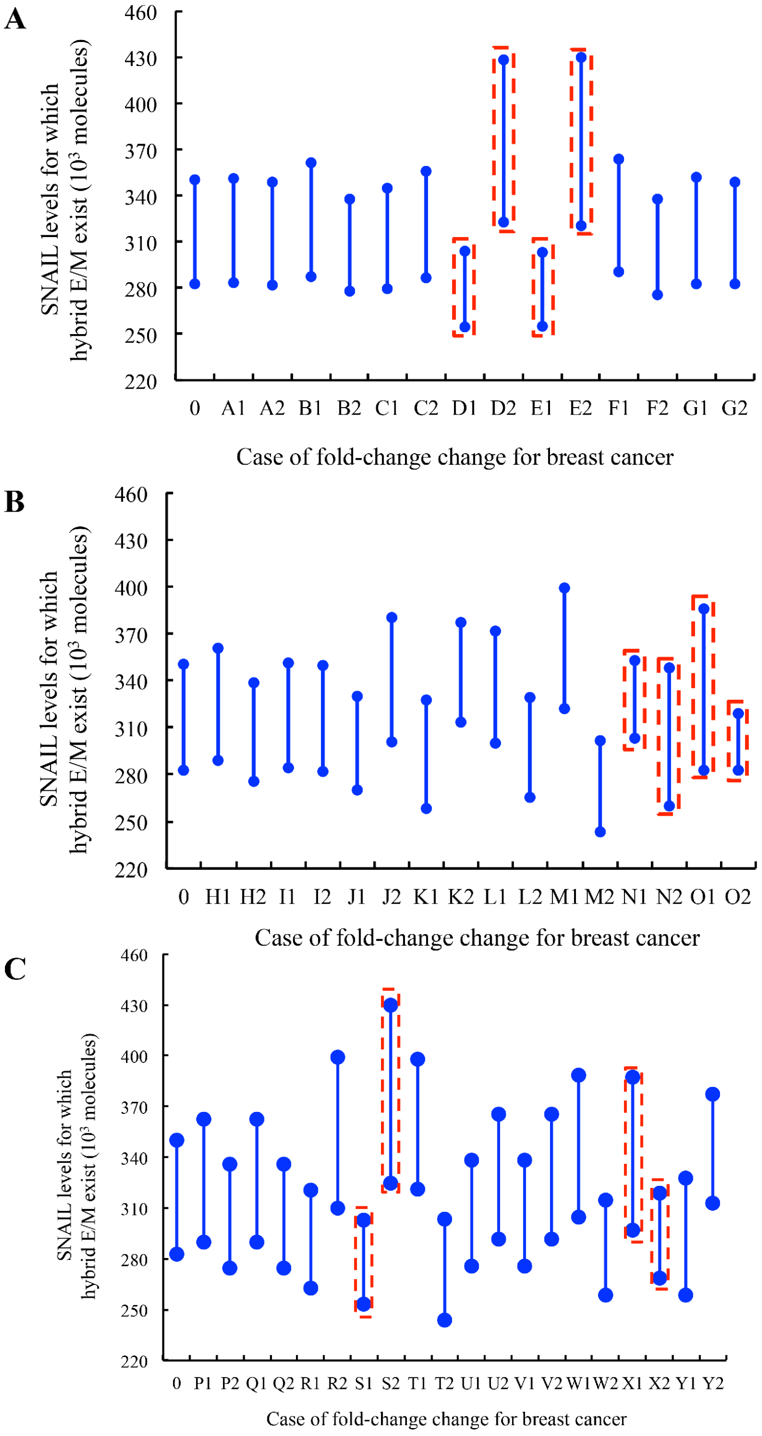

Supplementary Figure S2: Sensitivity analysis of miR-200/ZEB/OVOL circuit driven by SNAIL for breast cancer. Alphanumeric codes on the x-axis represent the cases of different changed parameters. 0 represents the case for parameters in Figure 3B (control case).

levels of miR-200 (depicted by μ_0). N1 and N2 represent the case for 20% increase and decrease in threshold levels of SNAIL for ZEB activation (depicted by $S_0^{m_Z}$). O1 and O2 represent the case for 20% increase and decrease in threshold levels of SNAIL for miR-200 inhibition (depicted by $S_0^{\mu_{200}}$). P1 and P2 represent the case for 20% increase and decrease in threshold levels of ZEB for its self-activation ($Z_0^{m_Z}$). Q1 and Q2 represent the case for 20% increase and decrease in threshold levels of ZEB for miR-200 inhibition (depicted by $Z_0^{\mu_{200}}$).

Figure S1C represent the cases for 20% increase and decrease in innate production rates and degradation rates

of different species. R1 and R2 represent the case for 20% increase and decrease of innate production rate of protein OVOL (depicted by g_O). S1 and S2 represent the case for 20% increase and decrease of innate production rate of OVOL mRNA (depicted by g_{m_O}). T1 and T2 represent the case for 20% increase and decrease of innate production rate of protein ZEB (depicted by g_Z). U1 and U2 represent the case for 20% increase and decrease of production rate of ZEB mRNA (depicted by g_{m_Z}). V1 and V2 represent the case for 20% increase and decrease of miR-200 production rate (depicted by $g_{\mu_{200}}$). W1 and W2 represent the case for 20% increase and decrease of innate degradation rate of protein OVOL (depicted by k_O). X1 and X2 represent the case for 20% increase and decrease of innate degradation rate of OVOL mRNA (depicted by k_{m_O}). Y1 and Y2 represent the case for 20% increase and decrease of innate degradation rate of protein ZEB (depicted by k_Z). Z1 and Z2 represent the case for 20% increase and decrease of innate degradation rate of ZEB mRNA (depicted by k_{m_Z}). A11 and A12 represent the case for 20% increase and decrease of miR-200 degradation rate (depicted by $k_{\mu_{200}}$).

Figure S2A represent the cases for 20% increase and decrease in the weight factors for different shifted Hill functions that represent fold-changes from the basal synthesis rate due to transcriptional regulation. A1 and A2 represent the case for 20% increase and decrease in fold-change for the self-inhibition of OVOL (depicted by λ_{O,m_O}). B1 and B2 represent the case for 20% increase and decrease in fold-change for the inhibition of OVOL by ZEB (depicted by λ_{Z,m_O}). C1 and C2 represent the case for 20% increase and decrease in fold-change parameter for the inhibition of ZEB by OVOL (depicted by λ_{O,m_Z}). D1 and D2 represent the case for 20% increase and decrease in fold change for the activation of ZEB by SNAIL (depicted by λ_{S,m_Z}). E1 and E2 represent the case for 20% increase and decrease in fold change parameter for the self-activation of ZEB (depicted by λ_{Z,m_Z}). F1 and F2 represent the case for 20% increase and decrease in fold change parameter for the inhibition of miR-200 by SNAIL (depicted by $\lambda_{S,\mu_{200}}$). G1 and G2 represent the case for 20% increase and decrease in the fold change parameters for the inhibition of miR-200 by ZEB (depicted by $\lambda_{Z,\mu_{200}}$).

Figure S2B represent the cases for 20% increase and decrease in the threshold levels of the different shifted Hill functions. H1 and H2 represent the case for 20% increase and decrease in threshold levels of OVOL for its self-inhibition (depicted by $O_0^{m_O}$). I1 and I2 represent the case for 20% increase and decrease in threshold levels of ZEB for OVOL inhibition (depicted by $Z_0^{m_O}$). J1 and J2 represent the case for 20% increase and decrease in threshold levels of OVOL for ZEB inhibition (depicted by $O_0^{m_Z}$). K1 and K2 represent the case for 20% increase and decrease in threshold levels of miR-200 (depicted by μ_0). L1 and L2 represent the case for 20% increase and decrease in threshold levels of SNAIL for ZEB activation (depicted by $S_0^{m_Z}$). M1 and M2 represent the case for 20% increase and decrease in threshold levels of SNAIL for miR-200 inhibition (depicted by $S_0^{\mu_{200}}$). N1 and N2 represent the case for 20% increase and decrease in threshold levels of ZEB for its self-activation ($Z_0^{m_Z}$). O1 and O2 represent the case for 20% increase and decrease in threshold levels of ZEB for miR-200 inhibition (depicted by $Z_0^{\mu_{200}}$).

Figure S2C represent the cases for 20% increase and decrease in innate production rates and degradation rates of different species. P1 and P2 represent the case for 20% increase and decrease of protein OVOL innate production rate (depicted by g_O). Q1 and Q2 represent the case for 20% increase and decrease of innate production rate of OVOL mRNA (depicted by g_{m_O}). R1 and R2 represent the case for 20% increase and decrease of innate production rate of protein ZEB (depicted by g_Z). S1 and S2 represent the case for 20% increase and decrease of ZEB mRNA production rate (depicted by g_{m_Z}). T1 and T2 represent the case for 20% increase and decrease of miR-200 production rate (depicted by $g_{\mu_{200}}$). U1 and U2 represent the case for 20% increase and decrease of the innate degradation rate of protein OVOL (depicted by k_O). V1 and V2 represent the case for 20% increase and decrease of innate degradation rate of OVOL mRNA (depicted by k_{m_O}). W1 and W2 represent the case for 20% increase and decrease of innate degradation rate of protein ZEB (depicted by k_Z). X1 and X2 represent the case for 20% increase and decrease of innate degradation rate of ZEB mRNA (depicted by k_{m_Z}). Y1 and Y2 represent the case for 20% increase and decrease of miR-200 degradation rate (depicted by $k_{\mu_{200}}$).

The effects of external noise on ZEB/OVOL circuit

Here, we investigate the dynamics of ZEB/OVOL circuit (Figure S3A). Dynamics of OVOL mRNA (m_O), protein OVOL (O), ZEB mRNA (m_Z) and protein ZEB (Z) can be described as:

$$\frac{dm_O}{dt} = g_{m_O} H^{S-}(Z, \lambda_{Z,m_O}) H^{S-}(O, \lambda_{O,m_O}) H^{S+}(S, \lambda_{S,m_Z}) - k_{m_O} m_O$$

$$\frac{dO}{dt} = g_O m_O - k_O O$$

$$\frac{dm_Z}{dt} = g_{m_Z} H^{S+}(Z, \lambda_{Z,m_Z}) H^{S-}(O, \lambda_{O,m_Z}) - k_{m_Z} m_Z$$

$$\frac{dZ}{dt} = g_Z m_Z - k_Z Z$$

We simulate the dynamics of ZEB/OVOL circuit with the external noise in the activation signal S of OVOL. To simulate this external noise, the signal S follows the stochastic differential equation:

$$\dot{S} = \alpha(S_0 - S) + \eta(t)$$

where $< \eta(t)\eta(t') > = \Gamma\delta(t - t')$. S_0 is set to be $80*10^3$ molecules, α is set to be 0.02 hour^{-1}, and Γ to be 16 (10^3

molecules)^2/hour. So the mean value of S is $S_0 = 80*10^3$ molecules, $E(\eta) = 0$, $\sigma_\eta = \sqrt{\Gamma/_{dt}} = 40$ (10^3 molecules/hour), where *dt* is chosen as 0.01 hour.

In our simulations, we considered this noise signal S as an input for the ZEB/OVOL circuit, and determined the corresponding variation in the levels of protein OVOL. We found that the self-inhibition of OVOL reduces this variation of protein OVOL levels (compare the black and red lines with blue and green lines). However, the self-activation of ZEB didn't affect the response of the circuit to external noise (compare the black and green lines with red and blue lines) (Figure S3B). Therefore, the self-inhibition of OVOL, but not self-activation of ZEB, may serve as a filter of the external noise, thus preventing aberrant triggering of EMT.

Nullclines of the miR-200/ZEB/OVOL circuit for the case of breast cancer

In Figure 2C, 2D, we show the nullclines of the miR-200/ZEB/OVOL circuit for prostate cancer under different SNAIL levels. At lower levels of SNAIL, the cells can be either in the epithelial - (high miR-200, low ZEB), or hybrid E/M - (medium miR-200, medium ZEB) phenotype (Figure 2C). However, at higher levels of SNAIL, the cells can be in hybrid E/M or mesenchymal – (low miR-200, high ZEB) state (Figure 2D). Here, we show the nullclines of miR-200/ZEB/OVOL circuit for breast cancer, i.e. when OVOL inhibits only ZEB but not miR-200 (Figure S4A–S4C). At different SNAIL levels, cells have different plasticity – they can adopt (a) only either epithelial or hybrid E/M (Figure S4A), or (b) only hybrid E/M (Figure S4B) or (c) only either hybrid E/M or mesenchymal (Figure S4C). Importantly, at SNAIL levels = $300*10^3$ molecules, the prostate cancer cells can obtain only the hybrid E/M stable state (Figure S4D), while breast cancer cells can also adopt epithelial phenotype in addition to the hybrid E/M phenotype (Figure S4C). Therefore, based on these results and simulations in Figure 3, we can infer that the 'expander' role of OVOL is more pronounced in the case of prostate cancer than for breast cancer.

Analyzing the miR-200/ZEB/OVOL circuit with different strengths of inhibition of OVOL by ZEB

Here, we investigate how the strength of inhibition of ZEB on OVOL affects the range of SNAIL levels for which hybrid E/M state can exist – for both prostate cancer and breast cancer. We calculate a phase diagram (two-dimensional bifurcation diagram) where the action of SNAIL is represented by two independent signals – S1, transcriptional activator of ZEB and S2, transcriptional inhibitor of miR-200 (Figure S5A-S5D). These diagrams demonstrate seven different phases (or sets of co-existing phenotypes for the same physiological conditions): three phases where cells can attain only phenotype - {E}, {M} and {E/M}, three phases where the cells can attain either of the two possible phenotypes – {E, M}, {E/M, M} and {E, E/M}, and one phase where the cells can attain any of the three possible phenotypes – {E, M, E/M}. We found that for circuits for both cases – prostate and breast cancer – lower inhibition of OVOL by ZEB leads to a larger range of physiological parameters for which the hybrid E/M phenotype can exist alone or as one of multiple possible phenotypes (compare the area bound by black dots in Figure S5B, D vs. that in Figure S5A, C). Therefore, the effect of OVOL as a promoter of the hybrid E/M phenotypes is amplified when it's weakly or not inhibited by ZEB.

To further validate the insights about how OVOL's inhibition by ZEB affects circuit dynamics, we calculate calculating a phase diagram for SNAIL and λ_{Z,m_O}(fold-change in OVOL's transcription rate due to repression by ZEB), when the prostate cancer

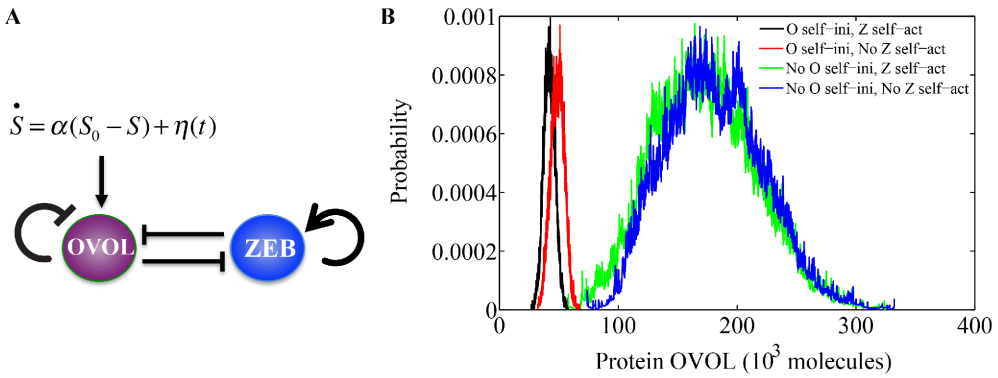

Supplementary Figure S3: ZEB/OVOL behaves as a filter for external noise. A. ZEB/OVOL mutual inhibitory circuit with OVOL self-inhibition and ZEB self-activation. The external noise is included in signal S, which is an input for the ZEB/OVOL circuit. **B.** The distribution of protein OVOL levels under different conditions - complete ZEB/OVOL circuit as shown in figure S3A (black line), ZEB/OVOL circuit without ZEB self-activation (red line), ZEB/OVOL circuit without OVOL self-inhibition (green line) and ZEB/OVOL circuit without ZEB self-activation and OVOL self-inhibition (blue line).

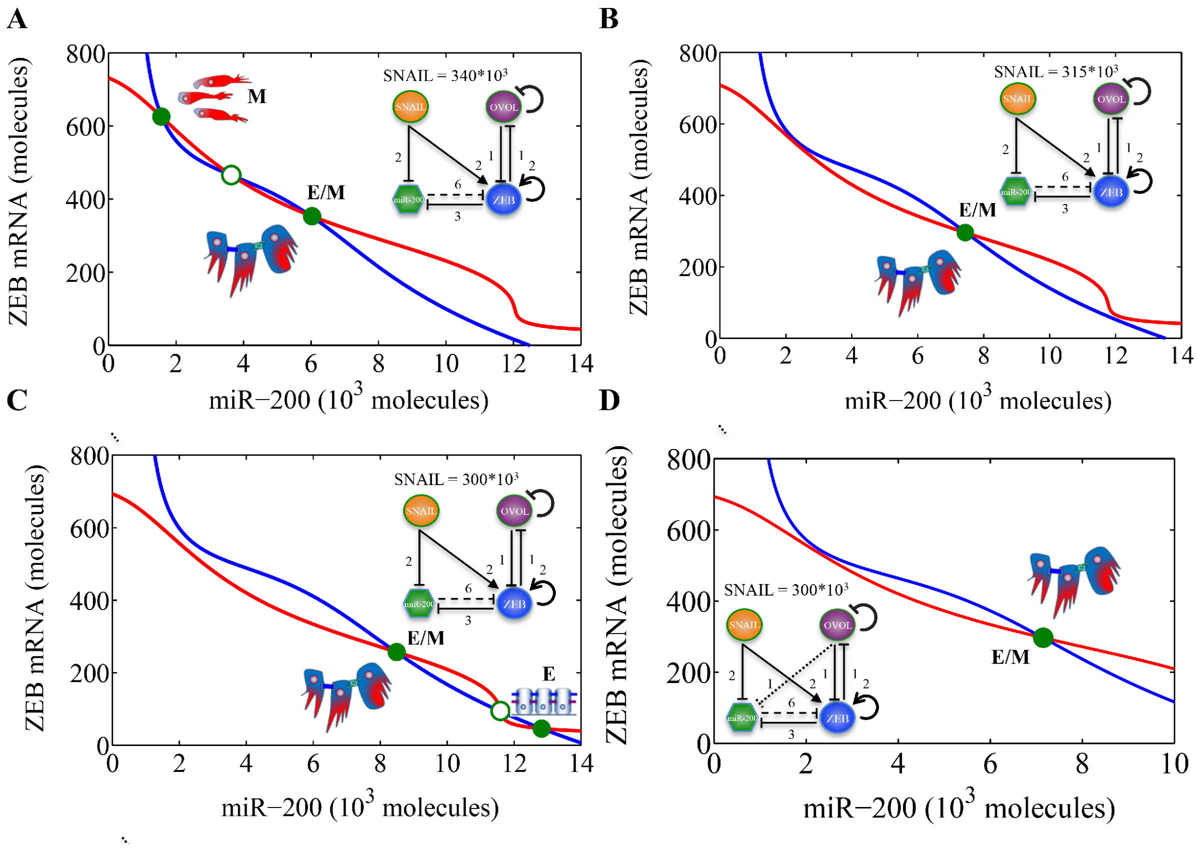

Supplementary Figure S4: Nullclines of miR-200/ZEB/OVOL circuit for breast cancer with different SNAIL levels. 340*10³ molecules **A.** 315*10³ molecules **B.** and 300*10³ molecules **C. D.** is the nullcline of miR-200/ZEB/OVOL circuit for prostate cancer with fixed SNAIL levels = 300*10³ molecules. Red nullcline is for the condition $dm_Z/dt = 0$, $dm_O/dt = 0$, $dZ/dt = 0$, $dO/dt = 0$ and blue nullcline for $d\mu_{200}/dt = 0$, $dm_O/dt = 0$, $dZ/dt = 0$, $dO/dt = 0$. Green solid circles denote stable fixed points, and green hollow circles denote unstable fixed points. Corresponding phenotypes have been depicted alongside the stable steady states.

circuit is driven by an external inhibition signal (SI) on OVOL (Figure S6A). We found that the {E/M} phase exists only when ZEB inhibits OVOL weakly (Figure S6B). Further, in phase diagrams of SNAIL and SI as the external signals, the {E/M} phase was seen only for a weak inhibition of OVOL by ZEB (Figure S6C, D), thus consistent with our results that OVOL acts as an 'expander' of the E/M phenotype, and our insights from sensitivity analysis of the model for miR-200/ZEB/OVOL circuit both for prostate and breast cancer.

Bifurcation diagram for the miR-200/ZEB/OVOL circuit driven by an external activation signal SA on OVOL

Here, we show how the miR-200/ZEB/OVOL circuit (both for prostate cancer and for breast cancer) reacts to external activation signal on OVOL, when the cell is initially in the epithelial phenotype or the hybrid E/M phenotype. For cells with very low levels of SNAIL, over-expression of OVOL does not cause any phenotypic transition, as the cells are already epithelial (Figure S7A, C). However, when cells are in the hybrid E/M phenotype for no external activation (or endogenous levels) of OVOL, the overexpression of OVOL induces a complete MET, both for the case of prostate cancer (Figure S7B) and breast cancer (Figure S7D).

Bifurcation for the miR-200/ZEB/OVOL circuit driven by an external inhibition signal SI on OVOL

At SNAIL levels corresponding to the hybrid E/M phenotype, inhibiting OVOL causes the cells transition to a mesenchymal phenotype, both in prostate and breast cancer (Figure 5A, C). Here, we investigate the response of miR-200/ZEB/OVOL circuit to external inhibition signal on OVOL when cells are epithelial or mesenchymal to begin with. We found that for both epithelial and mesenchymal cells, inhibition of OVOL does not cause a

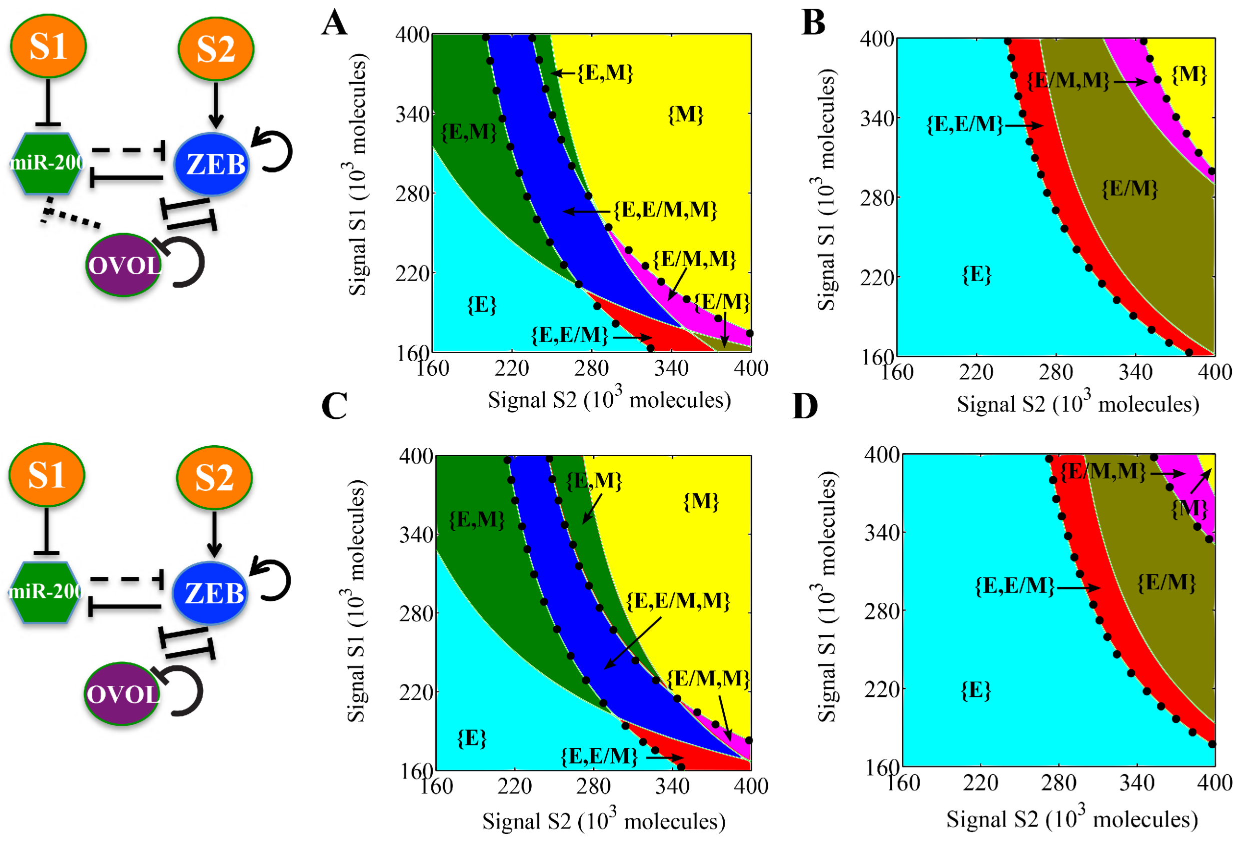

Supplementary Figure S5: Phase-diagrams of miR-200/ZEB/OVOL circuit with different strengths of inhibition of OVOL by ZEB. The phase diagrams in every row are for the circuit drawn in the leftmost column of that row - **A.** and **B.** for prostate cancer, **C.** and **D.** for breast cancer. $\lambda_{Z,m_o} < 1$ is the fold-change from basal synthesis rate of OVOL mRNA by binding of protein ZEB. Since ZEB transcriptionally inhibits OVOL, $<\lambda_{Z,m_o}$ 1. The smaller λ_{Z,m_o} is, the stronger the inhibition of ZEB on OVOL. Phase-diagram driven by two independent signals S1 and S2 representing SNAIL, for A $\lambda_{Z,m_o} = 0.1$ and miR-200/ZEB/OVOL circuit when OVOL inhibits both miR-200 and ZEB; B $\lambda_{Z,m_o} = 0.9$ and miR-200/ZEB/OVOL circuit when OVOL inhibits both miR-200 and ZEB; C $\lambda_{Z,m_o} = 0.1$ and miR-200/ZEB/OVOL circuit when OVOL inhibits only miR-200 but not ZEB; and D $\lambda_{Z,m_o} = 0.9$ and miR-200/ZEB/OVOL circuit when OVOL inhibits both miR-200 and ZEB. Different colors in A - D represent different phases (or set of co-existing phenotypes for the same physiological conditions). Area bound by the black dots shows the total range of physiological parameters for which the hybrid E/M phenotype exists, either alone or in combination with other possible phenotypes.

phenotype transition, both for the case of breast cancer and prostate cancer (Figure S8A-D).

Temporal dynamics of epithelial-hybrid-mesenchymal transitions in breast cancer

In presence of OVOL, both EMT and MET are two-step processes in the case of prostate cancer (Figure 5). Here we show the temporal dynamics of EMT in breast cancer (Figure S9). Similar to its role in prostate cancer, OVOL enables the cells to undergo partial MET in breast cancer as well (Figure S9B). Compared with temporal dynamics of EMT in prostate cancer (Figure 6C), the transition time point from E to hybrid E/M is about one day later and the transition time point from M to hybrid E/M is one day earlier in breast cancer (Figure S9D) with the same external signal SNAIL. The required levels of SNAIL to induce a transition from E to E/M is somewhat higher in breast cancer (SNAIL = 309*10^3 molecules, Figure 3B) than that in prostate cancer (SNAIL = 287*10^3 molecules, Figure 3C) and the required levels of SNAIL to induce a transition from M to E/M is also higher in breast cancer (SNAIL = 320*10^3 molecules, Figure 3B) than that in prostate cancer (SNAIL = 306*10^3 molecules, Figure 3C), so higher SNAIL levels are needed for breast cancer cells to undergo both EMT and MET vis-a-vis prostate cancer.

OVOL inhibition on SNAIL allows more plasticity

Recent unpublished work from Pienta group shows that OVOL can directly inhibit the transcription of SNAIL. However, details such as the number of binding sites are not well-known. To mimic the effects of this possible link, we weakened both the activation of SNAIL on ZEB and inhibition of SNAIL on miR-200 and analyzed the dynamics of the miR-200/ZEB/OVOL

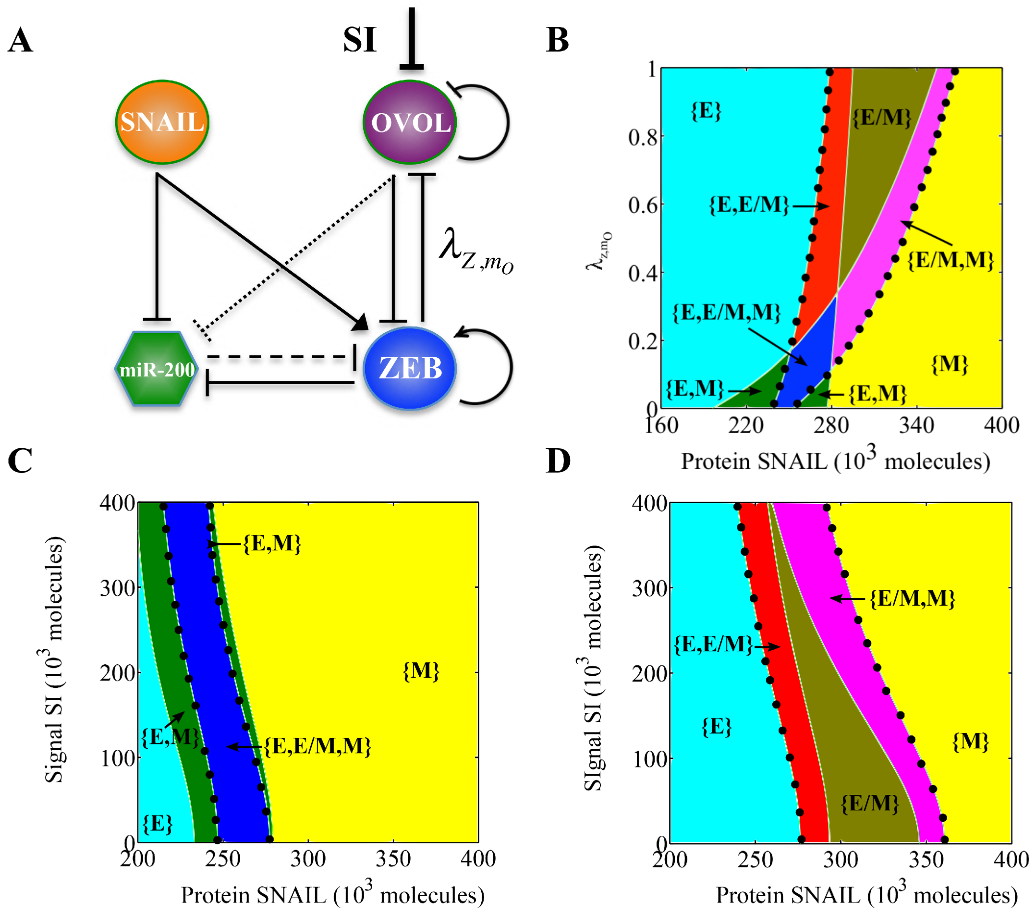

Supplementary Figure S6: Phase-diagrams of miR-200/ZEB/OVOL circuit for prostate cancer with different strengths of inhibition of OVOL by ZEB. A. miR-200/ZEB/OVOL circuit for prostate cancer. λ_{Z,m_O} is the fold-change from basal synthesis rate of OVOL mRNA by binding of protein ZEB. Since ZEB transcriptionally inhibits OVOL, $\lambda_{Z,m_O} < 1$. The smaller the λ_{Z,m_O}, the stronger the inhibition of ZEB on OVOL. **B.** Phase diagram of miR-200/ZEB/OVOL circuit driven by two parameters - λ_{Z,m_O} and SNAIL levels. **C.** Phase diagram of miR-200/ZEB/OVOL circuit with $\lambda_{Z,m_O} = 0.1$ driven by variable levels of both SNAIL and the external inhibition signal (SI) on OVOL. **D.** Phase diagram of miR-200/ZEB/OVOL circuit with $\lambda_{Z,m_O} = 0.9$ driven by variable levels of both SNAIL and the external inhibition signal (SI) on OVOL. Different colors in A - D represent different phases (set of co-existing phenotypes for the same physiological conditions). Area bound by the black dots shows the total range of physiological parameters for which the hybrid E/M phenotype exists, either alone or in combination with other possible phenotypes.

circuit for both breast cancer and prostate cancer. As a result, higher levels of SNAIL are required for the transition from E to E/M and E/M to M. In addition, the range of physiological parameters for which the hybrid E/M phenotype can exist alone or as one of multiple possible phenotypes increases is enlarged further when OVOL inhibition on SNAIL is included. Specifically, the increased existence of the hybrid E/M phenotype is larger for prostate cancer (when OVOL inhibits the miR-200) as compared to breast cancer (no or weak inhibition of miR-200 by OVOL) (compare the increase in green shaded region in Figure S10C vs that in Figure S10D, with the increase in the green shaded region in Figure S10B vs that in Figure S10A), which is consistent with our previous results. Hence, OVOL inhibition on SNAIL allows the miR-200/ZEB/OVOL circuit to allow a larger region for the existence of the hybrid E/M phenotype, thus increasing the plasticity for the cancer cells.

Bioinformatics analysis of OVOL regulation of EMT/MET

ZEB and OVOL inhibit each other in both breast cancer and prostate cancer cells [7]. Here, we analyzed and evaluated the correlations of ZEB and OVOL in both breast and prostate cancer tissue samples. From NCBI's Gene Expression Omnibus (GEO), expressions of genes OVOL1, OVOL2, ZEB1 (TCF8), ZEB2 (SIP1) in datasets GSE20194 (breast cancer) and GSE 25136 (prostate cancer) were extracted. The data was preprocessed with RMA method [16] and then normalized to have zero mean and unit variance. Linear regression analysis was conducted and expression correlations were shown for the above-mentioned genes.

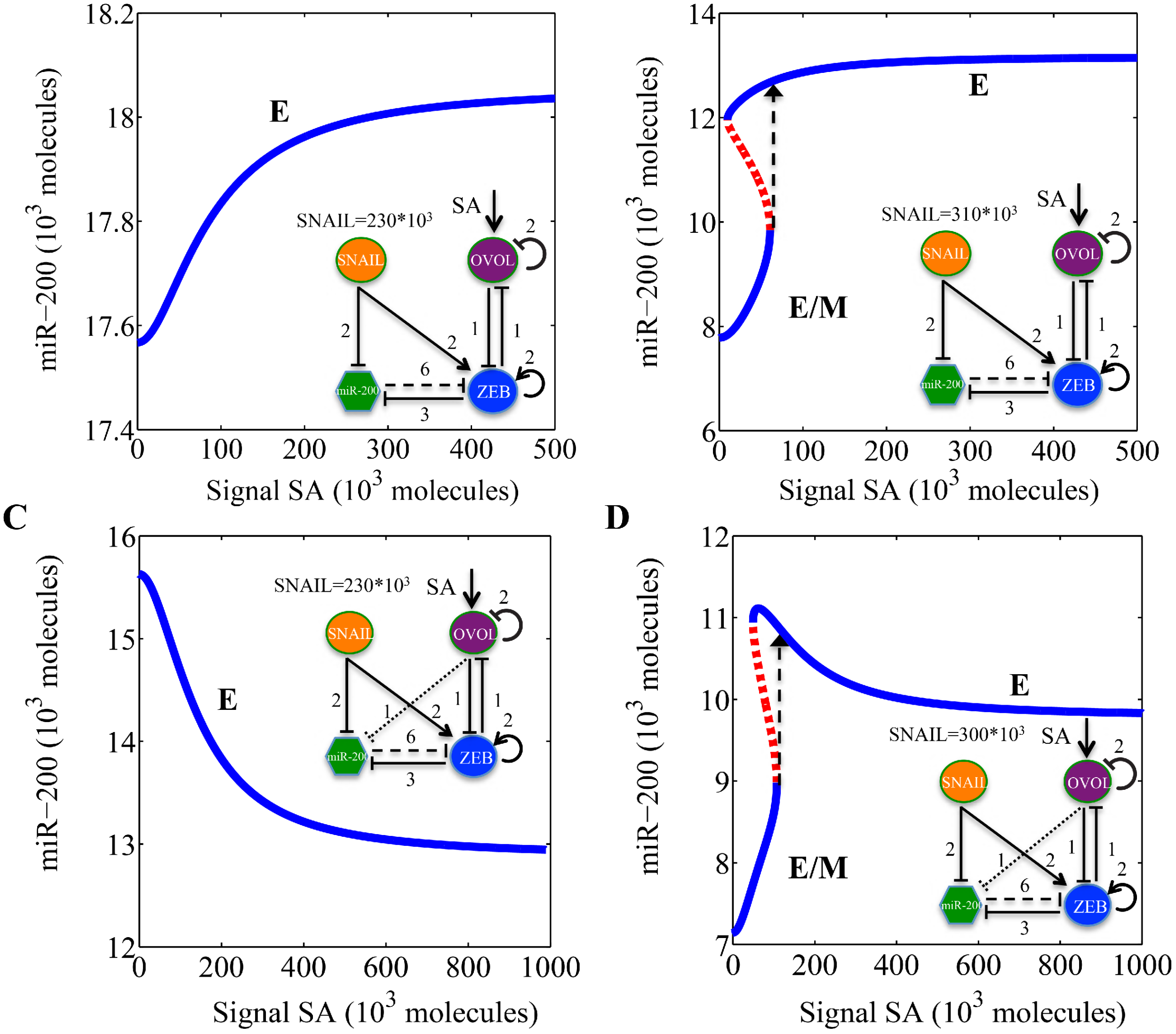

Supplementary Figure S7: Bifurcation for the miR-200/ZEB/OVOL circuit driven by an external activation signal (SA) on OVOL. **A.** and **B.** show the results of the circuit for the case of breast cancer (no inhibition of miR-200 by OVOL), and similarly **C.** and **D.** correspond to prostate cancer (inhibition of miR-200 by OVOL). **A.** Bifurcation of miR-200 levels when the cell is initially in epithelial phenotype (as enabled by SNAIL levels = 230×10^3 molecules) and is driven by varying activation signal (SA) on OVOL. **B.** Bifurcation of miR-200 levels when the cell is initially in hybrid E/M (as enabled by SNAIL levels = $310*10^3$ molecules) and is driven by varying activation signal (SA) on OVOL **C.** Bifurcation of miR-200 levels when the cell is initially in epithelial phenotype (enabled by SNAIL levels = 230×10^3 molecules) and is driven by varying activation signal (SA) on OVOL. **D.** Bifurcation of miR-200 levels when the cell is initially in hybrid E/M phenotype (as enabled by SNAIL levels = $300*10^3$ molecules) and is driven by varying activation signal (SA) on OVOL.

The negative correlation, although present for both tissues, between ZEB/OVOL seems to be tissue-specific – more for prostate cancer than for breast cancer (Figure S11). This insight from bioinformatics analysis is consistent with our results from mathematical modeling that although OVOL expands the region of existence for the E/M phenotype in both prostate and breast cancer; its effect is more pronounced for prostate cancer (Figure 3, Figure S4).

Finding OVOL targets through CHIP-Seq

OVOL1 and OVOL2 were amplified by PCR and cloned into pcDNA™-DEST47 (Life Technologies) to create c-terminal, GFP-tagged constructs. These constructs were then transfected into PC3-Epi cells [17] using X-tremeGENE HP DNA transfection reagent (Roche). 48 hours later, cells were washed with PBS, fixed with 1% formaldehyde in 1xPBS for 10 minutes, quenched

with 125 mM Glycine. Fixed cells were collected in 1xPBS by scraping and lysed with 1% SDS/5mM EDTA/50 mM Tris-HCL (pH 8.1) at 4°C with protease inhibitor cocktail (Roche). Extracts were then diluted 3 fold with 1% Triton X-100/2mM EDTA/150mM NaCl/20mM Tris-HCl (pH 8.1). Shearing was done using an M220 Focused-ultrasonicator (Covaris) until genomic DNA was approximately 150 bp average lengths. Immunoprecipiations were done with a GFP antibody ab290 (Abcam) and protein G coupled Dynabeads (Life Technologies). Washing of Dynabeads was sequential with TSE1 (0.1% SDS/1% Triton X-100/2mM EDTA/20mM Tris-HCl/150mM NaCl pH 8.1), TSE2 (0.1% SDS/1% Triton X-100/2mM EDTA/20mM Tris-HCl/500mM NaCl pH 8.1) and TSE3 (0.25M LiCl/1% NP-40/1% deoxycholate/1mM EDTA/10mM Tris-HCl pH 8.1). Extraction was done with 1% SDS/0.1M $NaHCO_3$/Proteinase K. DNA inputs and immunoprecipitations were sequenced using the HiSeq 2500 (Illumina). ChIP-seq data was analyzed using MACS [18].

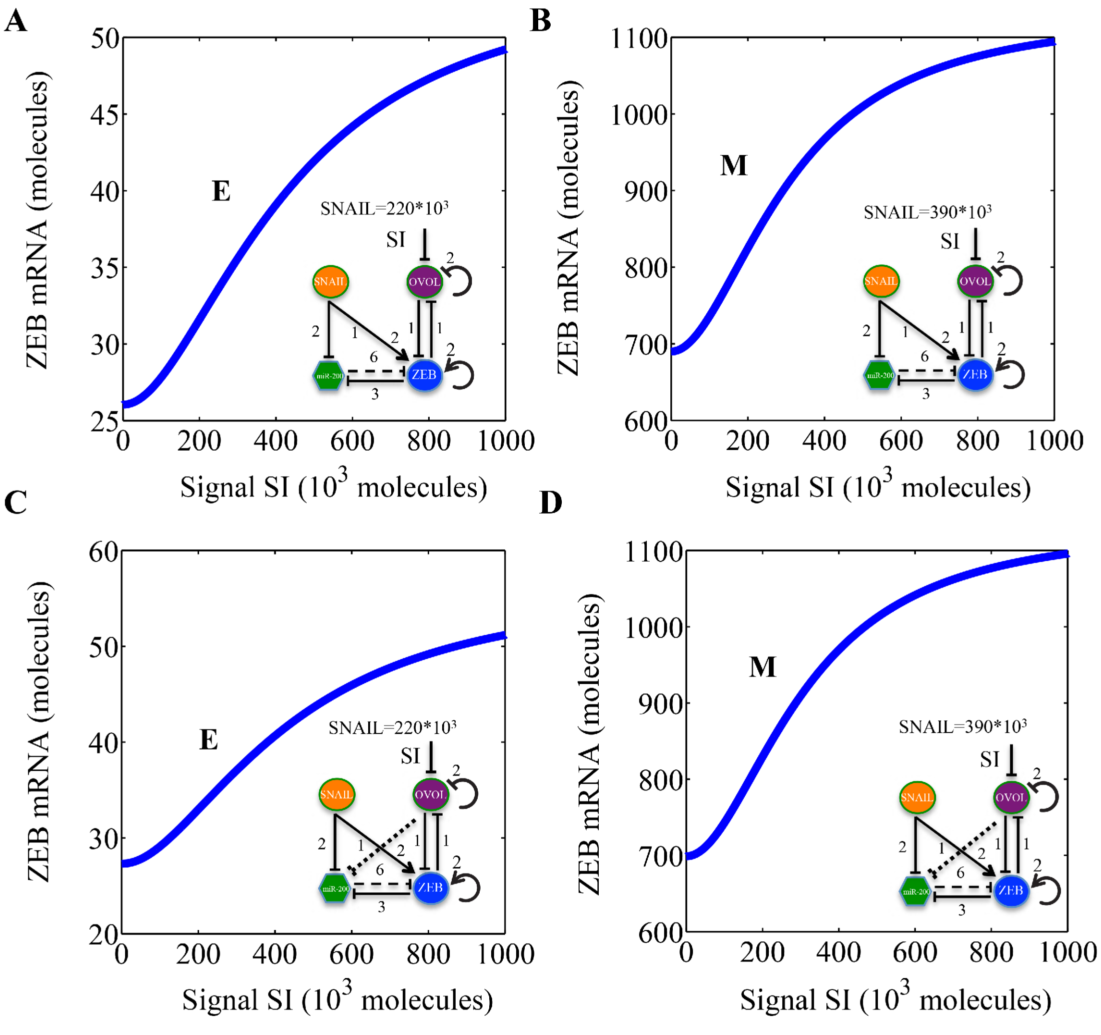

Supplementary Figure S8: Bifurcation for miR-200/ZEB/OVOL circuit in response to an external inhibition signal (SI) on OVOL. **A** and **B** show the results for the miR-200/ZEB/OVOL circuit for the case of breast cancer (no inhibition of miR-200 by OVOL), and **C** and **D** show corresponding results for the case of prostate cancer (OVOL inhibits both miR-200 and ZEB). **A.** Bifurcation of ZEB mRNA levels when the cell is initially in epithelial phenotype (as enabled by SNAIL levels = 220*10^3 molecules) and is driven by a varying inhibition signal (SI) on OVOL. SNAIL levels are fixed at. **B.** Bifurcation of ZEB mRNA levels when the cell is initially in mesenchymal phenotype (as enabled by SNAIL levels = 390*10^3 molecules) and is driven by a varying inhibition signal (SI) on OVOL. **C.** Bifurcation of ZEB mRNA levels when the cell is initially in epithelial phenotype (as enabled by SNAIL levels = 220*10^3 molecules) and is driven by a varying inhibition signal (SI) on OVOL. **D.** Bifurcation of ZEB mRNA levels when the cell is initially in mesenchymal phenotype (as enabled by SNAIL levels = 390*10^3 molecules) and is driven by a varying inhibition signal (SI) on OVOL.

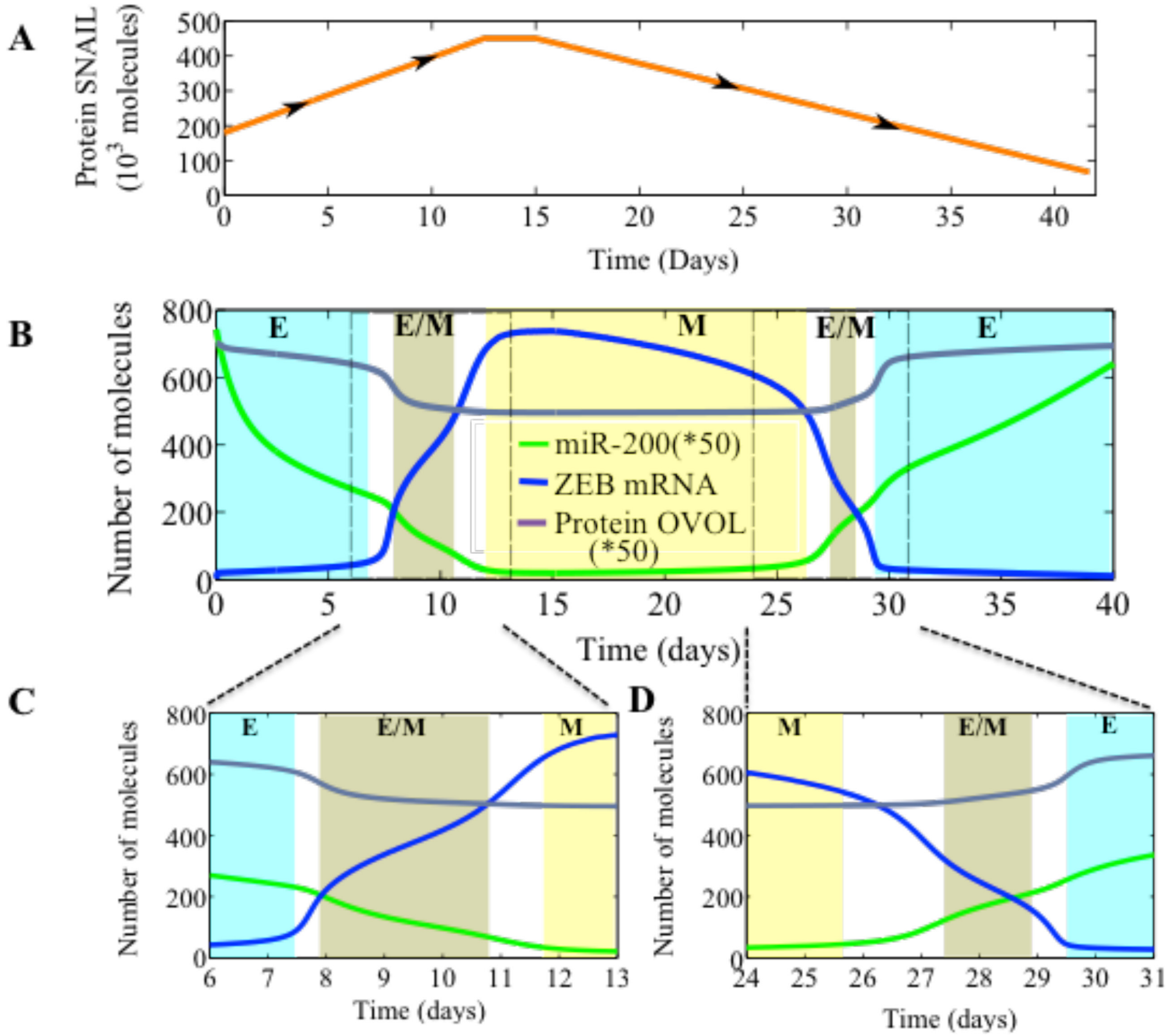

Supplementary Figure S9: Temporal dynamics of epithelial-hybrid-mesenchymal transitions in breast cancer. **A.** Time-varying external signal (SNAIL levels) applied to the miR-200/ZEB/OVOL circuit. **B.** Temporal evolution of miR-200 (green, scaled by 0.02 to fit in the plot), ZEB mRNA (blue) and protein OVOL (purple, scaled by 0.02 to fit in the plot) for the miR-200/ZEB/OVOL module. Areas shown in the boxes (days 5-12 and days 25-32) are expanded in **C.** and **D.** to show that the cells pass through the hybrid E/M state while undergoing EMT or MET, i.e. both EMT and MET are two-step processes – E->E/M->M, and M -> E/M -> E. Different colors in B-E represent different stable states or phenotypes - cyan for E or (1,0) state, brown for hybrid E/M or (½, ½) state, yellow for M or (0,1) state.

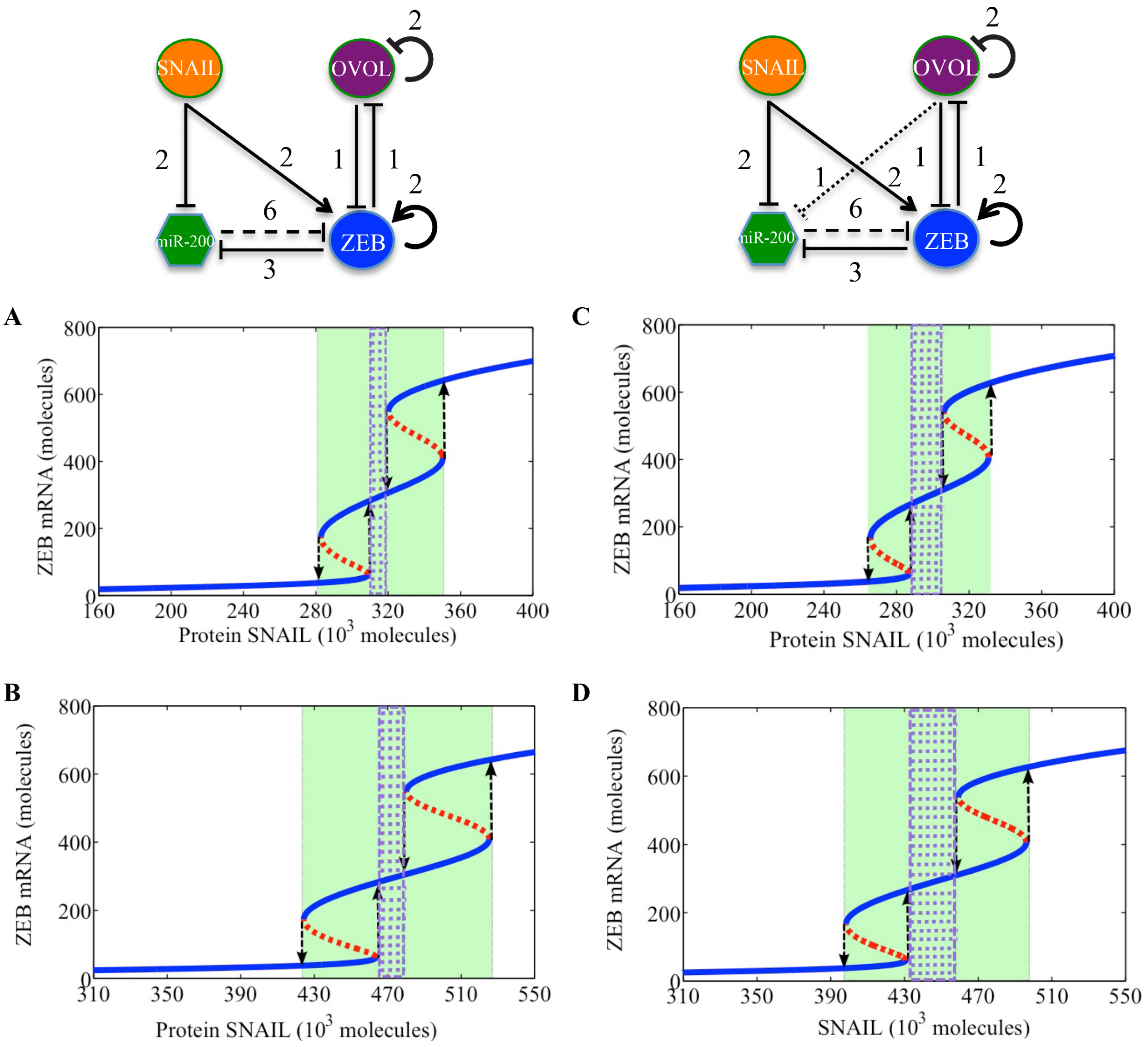

Supplementary Figure S10: Bifurcations for miR-200/ZEB/OVOL circuit driven by weaker interaction from SNAIL for both breast cancer (A and B) and prostate cancer (C and D) (See corresponding circuit diagrams at the top of the figure). For **A.** and **C.** $S^0_{m_z} = S^0_{\mu_{200}} = 180*10^3$ molecules; and for **B. D.** $S^0_{m_z} = S^0_{\mu_{200}} = 270*10^3$ molecules, where $S^0_{m_z}$ and $S^0_{\mu_{200}}$ are the respective thresholds for the Shifted Hill functions for the activation of SNAIL on ZEB and inhibition of SNAIL on miR-200. Larger value of the thresholds here means relatively weaker effects of SNAIL on miR-200 and ZEB, thus mimicking the inhibition of SNAIL by OVOL. Since SNAIL can self-inhibit, we increased $S^0_{m_z}$ and $S^0_{\mu_{200}}$ by 50% of its original values. . The range of SNAIL levels in all figures are kept the same - from $160*10^3$ molecules to $400*10^3$ molecules for A, C) and from $310*10^3$ molecules to $550*10^3$ molecules for B, D. The region marked in green represents the range of SNAIL levels for which the hybrid E/M phenotype can exist alone or as one of multiple possible phenotypes. The region marked by purple dots represents the range of SNAIL levels for which the only phenotype that the cells can adopt is the hybrid E/M one.

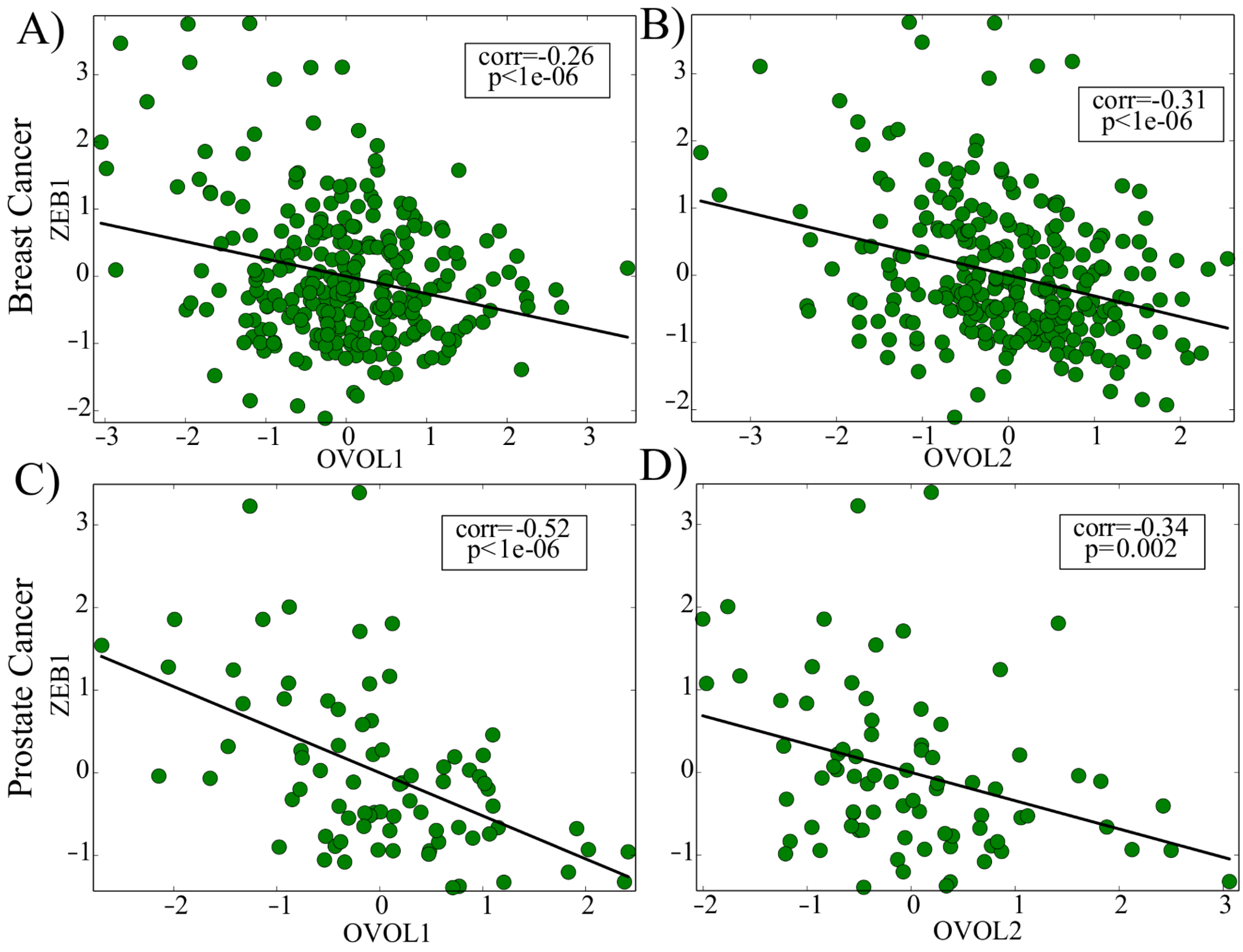

Supplementary Figure S11: Correlation between ZEB and OVOL in both breast cancer and prostate cancer tissue samples. A. B. represent the case for breast cancer – 230 samples (GSE20194); and **C. D.** represent that for prostate cancer – 79 samples (GSE25136). The variable *corr* represents the Pearson correlation between the variables and p represents the *p*-value. Black line represents the linear fit of the data. Each dot in the figure represents a sample. Samples with missing values and NANs were eliminated. We scaled the expression of $g_i^{'}$ using the formula $g_i^{'} = \frac{g_i - \bar{g}_i}{\sigma_i}$, where g_i is the expression of gene *i*, $\bar{g}_i$ is the mean expression of gene *i* and σ_i is the standard deviation of gene *i* from the relevant cancer patient data. The figure here shows the scaled expression levels of gene OVOL1, OVOL2 ZEB1 and ZEB2. *P*-value here shows the significance of statistically correlated expression. If *P*-value < 0.05, there is a significant correlation. If *P*-value > 0.05, there is no significant correlations.

REFERENCES

1. Lu M, Jolly M K, Levine H, Onuchic J N, Ben-Jacob E. MicroRNA-based regulation of epithelial-hybrid-mesenchymal fate determination. *Proc. Natl. Acad. Sci. U.S.A*. 2013; 110:18174–9.
2. Brabletz S, Brabletz T. The ZEB/miR-200 feedback loop—a motor of cellular plasticity in development and cancer? *EMBO Rep*. 2010; 11:670–7.
3. Hurteau G J, Carlson J A, Roos E. Stable expression of miR-200c alone is sufficient to regulate TCF8 (ZEB1) and restore E-cadherin expression. Cell Cycle. 2009; 8:2064–9.
4. Hill L, Browne G, Tulchinsky E. ZEB/miR-200 feedback loop: at the crossroads of signal transduction in cancer. *Int. J. Cancer*. 2013; 132:745–54.
5. Guaita S, Puig I, Franci C, Garrido M, Dominguez D, Batlle E, Sancho E, Dedhar S, De Herreros AG, Baulida J. Snail induction of epithelial to mesenchymal transition in tumor cells is accompanied by MUC1 repression and ZEB1 expression. *J. Biol. Chem*. 2002; 277:39209–16.
6. Kaller M, Hünten S, Menssen A, Götz U, Hermeking H, Siemens H, Jackstadt R. miR-34 and SNAIL form a double-negative feedback loop to regulate epithelial-mesenchymal transitions. *Cell Cycle*. 2011; 10:4256–71.
7. Roca H, Hernandez J, Weidner S, McEachin R C, Fuller D, Sud S, Schumann T, Wilkinson J E, Zaslavsky A, Li H, Maher C A, Daignault-Newton S, Healy P N, Pienta K J. Transcription Factors OVOL1 and OVOL2 Induce the Mesenchymal to Epithelial Transition in Human Cancer. *PLoS One*. 2013; 8:e76773.
8. Nair M, Bilanchone V, Ortt K, Sinha S, Dai X. Ovol1 represses its own transcription by competing with transcription activator c-Myb and by recruiting histone deacetylase activity. *Nucleic Acids Res*. 2007; 35:1687–97.
9. Schwanhäusser B, Busse D, Li N, Dittmar G, Schuchhardt J, Wolf J, Chen W, Selbach M. Global quantification of mammalian gene expression control. *Nature*. 2011; 473:337–42.
10. Eden E, Geva-Zatorsky N, Issaeva I, Cohen A, Dekel E, Danon T, Cohen L, Mayo A, Alon U. Proteome Half-Life Dynamics in Living Human Cells. *Science* (80-.). 2011; 331:764–8.
11. Yang E, van Nimwegen E, Zavolan M, Rajewsky N, Schroeder M, Magnasco M, Darnell JE. Decay rates of human mRNAs: correlation with functional characteristics and sequence attributes. *Genome Res*. 2003; 13:1863–72.
12. Gantier M P, McCoy C E, Rusinova I, Saulep D, Wang D, Xu D, Irving A T, Behlke MA, Hertzog P J, Mackay F, Williams B R G. Analysis of microRNA turnover in mammalian cells following Dicer1 ablation. *Nucleic Acids Res*. 2011; 39:5692–703.
13. Khanin R, Vinciotti V. Computational modeling of post-transcriptional gene regulation by microRNAs. *J. Comput. Biol. AJ. Comput. Mol. Cell Biol*. 2008; 15:305–16.
14. Milo R, Jorgensen P, Moran U, Weber G, Springer M. BioNumbers–the database of key numbers in molecular and cell biology. *Nucleic Acids Res*. 2010; 38:D750–3.
15. Lim L P, Lau N C, Weinstein EG, Abdelhakim A, Yekta S, Rhoades M W, Burge CB, Bartel DP. The microRNAs of Caenorhabditis elegans. *Genes Dev*. 2003; 17:991–1008.
16. Irizarry R A, Hobbs B, Beazer-barclay Y D, Antonellis K J, Scherf U W E, Speed T P. Exploration , normalization , and summaries of high density oligonucleotide array probe level data. *Biostatistics*. 2003; :249–64.
17. Mooney S M, Parsana P, Hernandez J R, Liu X, Verdone J E, Torga G, Harberg G, Pienta K J. The Presence of Androgen Receptor Elements Regulates ZEB1 Expression in the Absence of Androgen Receptor. *J. Cell. Biochem*. 2015; 116:115–23.
18. Zhang Y, Liu T, Meyer Ca, Eeckhoute J, Johnson DS, Bernstein B E, Nusbaum C, Myers R M, Brown M, Li W, Liu X S. Model-based analysis of ChIP-Seq (MACS). *Genome Biol*. 2008; 9–R137.
